# Cytomegalovirus immunity in high-risk liver transplant recipients following preemptive antiviral therapy versus prophylaxis

**DOI:** 10.1172/jci.insight.180115

**Published:** 2024-09-24

**Authors:** Danniel Zamora, Sayan Dasgupta, Terry Stevens-Ayers, Bradley Edmison, Drew J. Winston, Raymund R. Razonable, Aneesh K. Mehta, G. Marshall Lyon, Michael Boeckh, Nina Singh, David M. Koelle, Ajit P. Limaye

**Affiliations:** 1 Division of Allergy and Infectious Diseases, Department of Medicine, University of Washington, Seattle, Washington, USA.; 2 Vaccine and Infectious Disease Division, Fred Hutchinson Cancer Center, Seattle, Washington, USA.; 3 Division of Infectious Diseases, UCLA Medical Center, Los Angeles, California, USA.; 4 Division of Public Health, Infectious Diseases, and Occupational Medicine, Mayo Clinic, Rochester, Minnesota, USA.; 5 Division of Infectious Diseases, Emory University School of Medicine, Atlanta, Georgia, USA.; 6 Department of Medicine, University of Pittsburgh, Pittsburgh, Pennsylvania, USA.; 7 Transplant Infectious Diseases, VA Pittsburgh Healthcare System, Pittsburgh, Pennsylvania, USA.; 8 Department of Global Health and; 9 Department of Laboratory Medicine and Pathology, University of Washington, Seattle, Washington, USA.; 10 Benaroya Research Institute, Seattle, Washington, USA.

**Keywords:** Infectious disease, Transplantation, Cellular immune response, Drug therapy

## Abstract

CMV-specific T cells, NK cells, and neutralizing antibodies (nAbs) were assessed in a randomized trial of CMV prevention with preemptive antiviral therapy (PET) versus prophylactic antiviral therapy (PRO) in donor-seropositive/recipient-seronegative (D^+^R^–^) liver transplant recipients (LTxR) at 100 days (end of intervention) and at 6 and 12 months after transplant. The PET group had significantly increased numbers of circulating polyfunctional T cells, NK cells, and nAbs compared with the PRO group at day 100, and several CMV immune parameters remained significantly higher by 12 months after transplant. Among PET recipients, preceding CMV viremia (vs. no preceding viremia) was associated with significantly higher levels of most CMV immune parameters at day 100. Higher numbers of CMV-specific polyfunctional T cells and NKG2C^+^ NK cells at day 100 were associated with a decreased incidence of CMV disease in multivariable Cox regression. The strongest associations with protection against CMV disease were with increased numbers of CMV-specific polyfunctional CD4^+^ T cells, CD3^neg^CD56^dim^CD57^neg^NKG2C^pos^ cells, and CD3^neg^CD56^dim^CD57^pos^NKG2C^pos^ NK cells. Our results suggest that PET is superior to PRO for CMV disease prevention by allowing low-level CMV replication and associated antigen exposure that is promptly controlled by antiviral therapy and facilitates enhanced CMV protective immunity in D^+^R^–^ LTxR.

## Abstract 2

Cytomegalovirus(CMV)-specific immunity was assessed in adult CMV high-risk liver transplant recipients from a randomized controlled trial of preemptive antiviral therapy vs. prophylactic antiviral therapy.

## Introduction

CMV disease remains an important cause of morbidity and mortality in solid organ transplant recipients (SOTr) despite current preventive, diagnostic, and treatment strategies. The risk for CMV infection and disease is highest in CMV-seronegative recipients who receive an organ from a seropositive donor (D^+^R^–^); this population comprises approximately 20%–25% of all organ transplant recipients but account for approximately 90% of all CMV disease (1). CMV D^+^R^–^ status, independent of CMV disease, remains independently associated with worse long-term allograft and patient survival and is thought to be mediated by adverse effects of long-term subclinical CMV replication (2). The association of CMV with worse transplant outcomes/complications (acute allograft rejection, worse allograft or patient survival) has been termed “indirect effects” of CMV to highlight that these worse outcomes linked to CMV may occur even without clinically recognized CMV disease (i.e., that these adverse outcomes might be related to latent or subclinical CMV infection). Additionally, the proportion of CMV D^+^R^–^ transplants is significantly increasing for all organ types (3). Thus, optimizing immune control of CMV among D^+^R^–^ SOTr is a high priority to improve both short- and long-term outcomes in organ transplant recipients.

The two approaches for CMV disease prevention in SOTr are preemptive antiviral therapy (PET) and prophylactic antiviral therapy (PRO) (1). In PRO, patients at risk for CMV infection/disease (i.e., D^+^ or R^+^) receive an antiviral drug, and the goal is complete viral suppression for a prespecified duration after SOT (~3–6 months vs. longer following lung transplantation). In contrast, PET allows for low-grade viral replication during the period of most intense immunosuppression as monitored with a sensitive marker (typically with CMV viremia by qPCR). In PET, antiviral therapy is initiated only when early CMV replication is detected with the goal of preventing its progression to higher level replication and/or CMV disease (1). Each CMV prevention strategy has potential advantages and disadvantages (1, 4, 5). The length of PRO is typically limited by duration-dependent drug toxicities, costs, drug interactions, and/or risk for resistance to currently available antiviral agents. Delayed-onset CMV disease (after antiviral prophylaxis is discontinued) is common with PRO (especially in D^+^R^–^ patients) and has been independently associated with mortality (6–8). Conversely, PET has consistently been associated with lower rates of delayed-onset CMV disease (9–12). However, there are logistical concerns with PET such as frequent CMV monitoring and coordination of prompt initiation of antiviral therapy (13).

PRO has been the dominant CMV prevention strategy compared with PET in high-risk D^+^R^–^ SOTr in the United States, but its use is limited by drug toxicities, cost, resistance, and high rates of postprophylaxis CMV disease. In order to assess the relative efficacy of the two CMV prevention strategies on CMV disease and other clinical outcomes, we conducted a multicenter randomized NIH-sponsored trial (CMV Antiviral Prevention Strategies In D^+^R^–^ Liver Transplants [CAPSIL]) that directly compared the two strategies (14). D^+^R^–^ adult liver transplant recipients (LTxR) were randomized 1:1 to receive either PET or PRO with valganciclovir for 100 days. We demonstrated that PET significantly reduced the incidence of endpoint committee–adjudicated CMV disease by 1 year after transplant compared with PRO, from 19% to 9% (14). The mechanism underlying the observed reduction in CMV disease with PET versus PRO was hypothesized to be enhanced CMV-specific immune responses facilitated through greater antigen exposure during viral replication with PET, as previously suggested (9–12). This was supported by a preliminary analysis of after intervention (i.e., day 100) measurements of CMV-specific T cells and neutralizing antibody (nAb) (14). The goal of the present study was to conduct a more comprehensive longitudinal assessment of CMV immune responses between the two study arms and to assess the association of these immune responses as potential immune correlates of CMV disease by 1 year after transplant.

A body of evidence links polyfunctional CMV-specific T cell immunity with protection against CMV infection/disease in SOTr (15–18). Alternatively, NK cells have also been linked to immune control of CMV (19), and genetic deficiencies in NK cell immunity are associated with the development of severe herpesvirus infections (20). NK cells that express NKG2C expand following CMV infection and higher levels of NKG2C-expressing NK cells have been associated with control of CMV in kidney transplant recipients (19, 21–23). It has been proposed that NK cells that coexpress NKG2C and CD57 represent a more antigen-experienced subset of NKG2C-expressing NK cells that clonally expanded in response to CMV infection and may also be important in protective immunity. Thus, measuring CMV-specific polyfunctional T cells and NKG2C-expressing NK cell subsets longitudinally allowed us to further investigate the “immunologic thumbprint” of primary CMV infection in the D^+^R^–^ organ transplant setting.

The role of humoral immunity for protection against CMV is less clear. nAbs are presumed to be important in control of primary CMV infection (as in the case of D^+^R^–^ SOTr), and in vitro studies have shown that antibodies against the CMV pentameric complex are highly neutralizing and potent (24, 25). This has renewed interest in pentameric complex as a potential CMV vaccine antigen candidate (26–31). In a phase II randomized clinical trial of a CMV-specific monoclonal antibody with activity against pentameric complex in D^+^R^–^ kidney transplant recipients, there was a decreased risk of CMV disease (but not CMV infection) in monoclonal antibody recipients (32). Collectively, these findings suggest a potential protective role of nAbs in primary CMV infection following SOT or hematopoietic stem cell transplant (HSCT).

The primary objective of this study was to leverage the large multicenter randomized trial design, the endpoint committee–adjudicated clinical outcome (CMV disease), and prospective longitudinally collected samples from the CAPSIL study to compare CMV-specific T cell, NK cell, and nAb responses at 100 days, 6 months, and 12 months after transplant among CMV D^+^R^–^ LTxR randomized to either PET or PRO. The secondary objective was to test the hypothesis that PET preferentially facilitates CMV protective immunity by providing antigen exposure during controlled viral replication. An exploratory objective was to determine the relationship of each measured immune parameter at day 100 with the subsequent risk of late-onset CMV disease.

## Results

## Study population

Of the 205 randomized CMV D^+^R^–^ LTxRs in the original trial (NCT01552369), 152 (74%) had samples available for immune function testing at 100 days after transplant. The reasons for patient and sample exclusion are listed in Supplemental Figure 1 (supplemental material available online with this article; https://doi.org/10.1172/jci.insight.180115DS1). Baseline characteristics of patients included in the current study were similar to those for participants in the CAPSIL trial (Table 1). Seventy-three PET and 79 PRO recipients were included in the current study, and patient characteristics were also similar to those in the original trial within each treatment group (NCT01552369) (Supplemental Table 1). Twenty-one patients developed endpoint committee–adjudicated CMV disease by 12 months after transplant. Three patients developed CMV disease before day 100 after transplant and were excluded from the analyses of the association of day 100 posttransplant CMV immunity measures and delayed-onset CMV. The remaining 18 patients developed delayed-onset CMV at a median of 147 days after transplant (IQR, 142–173 days after transplant).

### T cell, NK cell, and nAb immune responses in those randomized to PET or PRO

#### Antigen-experienced T cells are increased following PET based on the expression of CD57.

**Figure 1:**
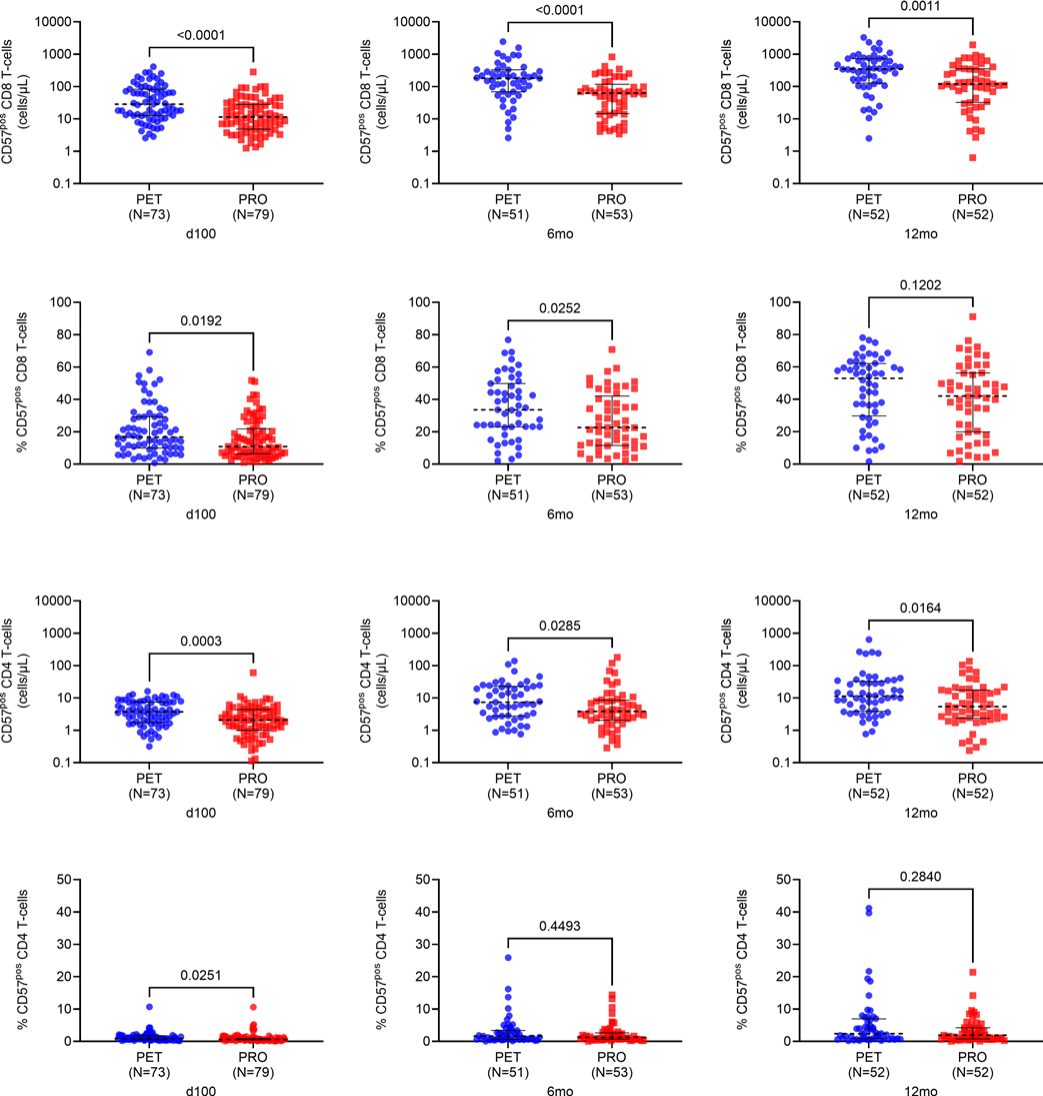
Absolute counts and proportions of antigen-experienced T cells at 100 days, 6 months, and 12 months after transplant based on the expression of CD57. CD8^+^ and CD4^+^ T cells were described as antigen-experienced based on cell surface level expression of CD57. CD57^+^ T cells were measured under nonstimulated testing conditions and are shown in PET vs. PRO recipients at all 3 time points. For absolute cell counts, 0 values were imputed as a low value (i.e., less than minimum of distribution) for graphing purposes owing to logarithmic scale conversion. Dotted black lines represent median values, and whiskers represent interquartile range. Comparisons were made using 2-sided Wilcoxon’s rank-sum testing at 95% CI.

Multiple T cell and NK cell subsets were evaluated using flow cytometry, and a representative gating scheme for each is shown in Supplemental Figure 2. We first compared absolute numbers of CD57-expressing antigen-experienced CD8^+^ and CD4^+^ T cells between treatment arms (Figure 1). CD57^+^ CD8^+^ and CD4^+^ T cell counts were significantly higher at 100 days (*P* < 0.001 and *P* = 0.0003, respectively), 6 months (*P* < 0.0001 and *P* = 0.03, respectively), and 12 months (*P* = 0.001 and *P* = 0.02, respectively) after transplant in the PET versus PRO groups. Similarly, the proportions of CD57^+^ CD8^+^ and CD4^+^ T cells were higher at 100 days (*P* = 0.02 and *P* = 0.03, respectively) in PET versus PRO recipients. However, only the proportion of CD57^+^ CD8^+^ T cells (but not CD4^+^ T cells) remained statistically higher in the PET group versus PRO group at 6 months after transplant (*P* = 0.03). These data demonstrate that PET is associated with a greater early expansion of antigen-experienced T cells based on the expression of CD57 with PET compared with PRO.

#### CMV-specific polyfunctional T cell responses are higher with PET versus PRO.

**Figure 2:**
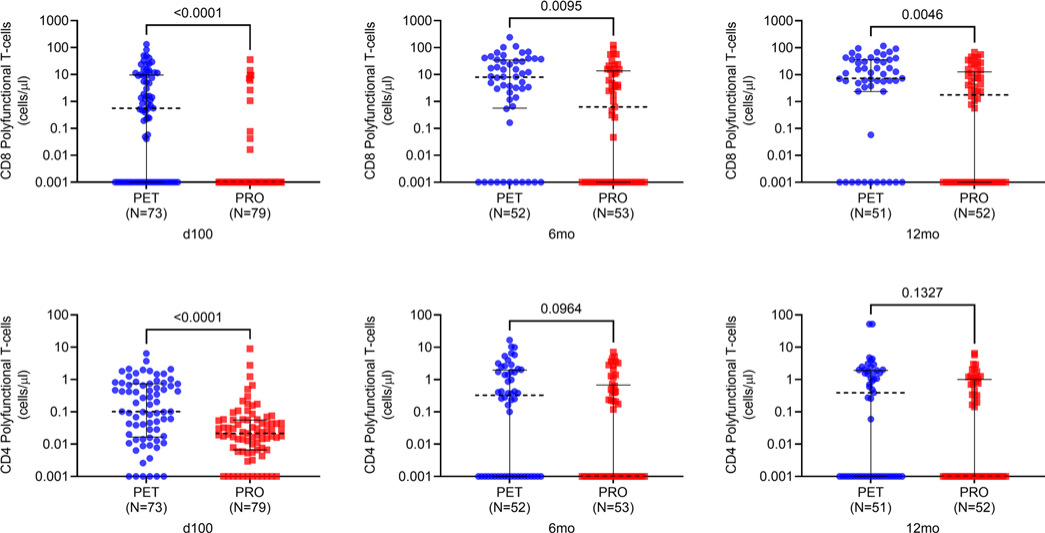
Absolute polyfunctional T cell counts following stimulation with CMV phosphoprotein 65. Absolute polyfunctional CMV-specific T cell counts based on the expression of IFN-γ plus at least 1 additional functional marker at 100 days, 6 months, and 12 months after transplant following stimulation with CMV phosphoprotein 65 (pp65) overlapping peptide library. For absolute cell counts, 0 values were imputed as a low value (i.e., less than minimum of distribution) for graphing purposes owing to logarithmic scale conversion. Dotted black lines represent median values, and whiskers represent interquartile range. Comparisons were made using 2-sided Wilcoxon’s rank-sum testing at 95% CI.

To assess CMV-specific polyfunctional T cell immunity following PET versus PRO, we compared absolute counts of CMV-specific polyfunctional T cells based on expression of IFN-γ plus at least 1 additional functional marker following stimulation with overlapping peptide pools of phosphoprotein 65 (pp65), an immunodominant CMV antigen (Figure 2). CMV-specific polyfunctional CD8^+^ T cell counts were higher in PET versus PRO recipients at 100 days (*P* < 0.001), 6 months (*P* = 0.005), and 12 months (*P* = 0.003) after transplant. Absolute CMV-specific polyfunctional CD4^+^ T cell counts were significantly higher in PET versus PRO recipients at 100 days after transplant (*P* < 0.001) but not at later time points. These data demonstrate that CMV-specific polyfunctional CD8^+^ (but not CD4^+^) T cells are higher with PET compared with PRO and remain significantly higher at 12 months after transplant.

We also compared the relative proportions of CMV-specific polyfunctional T cells stratified by the degree of their polyfunctionality based on the expression of IFN-γ plus at least 1 additional functional marker in response to stimulation with CMV pp65 peptide library (Supplemental Figure 3). Overall, the proportions of CMV-specific 2-, 3-, and 4-functional CD8^+^ T cell responses were similar in the PET versus PRO groups at all time points; whereas, CMV-specific polyfunctional CD4^+^ T cell responses were higher degree (i.e., 3-, 4-, and 5-functional) in the PET vs. PRO group at 6 and 12 months. Importantly, PET had a higher proportion of positive responses compared with PRO in all analyses. Therefore, unbiased evaluation of non–IFN-γ expressing CMV-specific polyfunctional T cell subsets may reveal important differences between groups.

#### CMV-specific polyfunctional T cell responses are higher with PET when assessed by the integrated COMPASS score.

To reduce the highly dimensional ICS data into meaningful summary statistics, we used the analytical combinatorial polyfunctionality analysis of antigen specific T cell subsets (COMPASS) package to generate T cell polyfunctionality scores (PFSs) and functionality scores (FSs).

**Figure 3:**
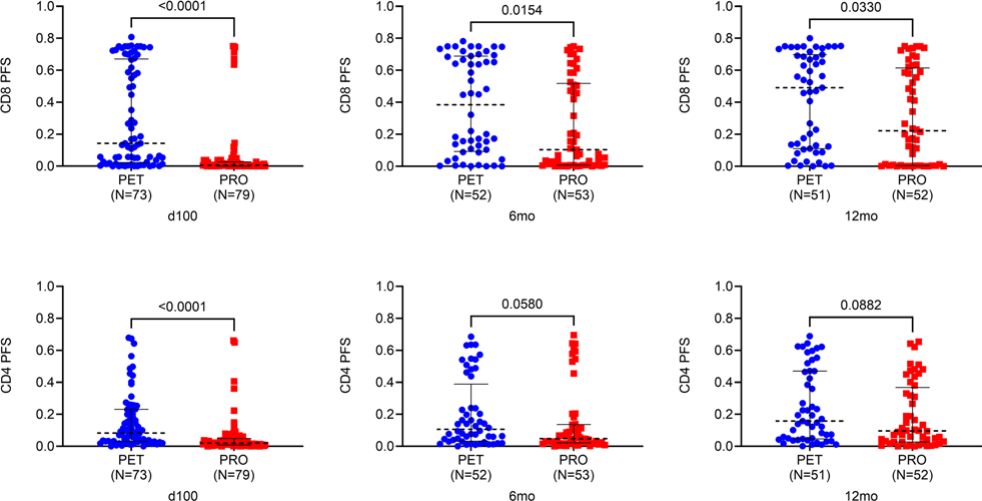
COMPASS polyfunctionality scores following stimulation with CMV phosphoprotein 65. COMPASS polyfunctionality scores (PFSs) at 100 days, 6 months, and 12 months after transplant following stimulation with CMV phosphoprotein 65 (pp65) overlapping peptide library. Patients were grouped according to treatment arm: PET (blue) vs. PRO (red). Dotted black lines represent median values, and whiskers represent interquartile range. Comparisons were made using 2-sided Wilcoxon’s rank-sum testing at 95% CI.

PFS differs from FS in that it weights T cell subsets by the degree of their polyfunctionality (i.e., cell subsets that respond to antigen with a greater number of markers receive larger weight), and both have been used to identify immune correlates in previous studies (33, 34). COMPASS scores were compared between treatment arms at day 100, 6 months, and 12 months after transplant (Figure 3). CD8 PFSs were increased in PET recipients compared with PRO recipients at 100 days (*P* < 0.001), 6 months (*P* = 0.02), and 12 months (*P* = 0.03) after transplant. CD4 PFSs were significantly increased in PET versus PRO recipients at 100 days after transplant only (*P* < 0.001), and they were numerically but not statistically higher at 6 and 12 months. Similar marked associations were seen with COMPASS FSs (data not shown). Notably, there were no differences in polyfunctional CD8^+^ or CD4^+^ T cell immunity by COMPASS following stimulation with our positive control test antigen, *Staphylococcal* enterotoxin B (SEB; data not shown). Thus, the COMPASS-integrated measures of the CMV-specific polyfunctional T cell response were higher with PET compared with PRO at the end of the CMV prevention intervention and persisted at 1 year after transplant for some of these measures.

#### NKG2C-expressing adaptive NK cells are numerically and proportionally increased with PET versus PRO.

NKG2C-expressing NK cells are increased in patients with CMV infection, and coexpression of CD57 in these cells indicates a more antigen-experienced subset (35, 36). Therefore, we compared NKG2C-expressing adaptive NK cell subsets based on cell surface level expression of CD56 (i.e., bright vs. dim) and CD57 (i.e., positive vs. negative). Specifically, we focused on NKG2C-expressing NK cell phenotypes based on the combination of these markers (from least to most antigen-experienced): CD3^neg^CD56^bright^CD57^neg^NKG2C^pos^, CD3^neg^CD56^dim^CD57^neg^NKG2C^pos^, and CD3^neg^CD56^dim^CD57^pos^NKG2C^pos^. The proportions of these NK cell types in PET versus PRO recipients at all 3 time points are shown in Supplemental Figure 4. Proportions of CD3^neg^CD56^dim^CD57^neg^NKG2C^pos^ and CD3^neg^CD56^dim^CD57^pos^NKG2C^pos^ NK cells were significantly increased in the PET versus PRO group at 100 days after transplant (*P* = 0.003 and *P* = 0.006, respectively), and the proportion of CD3^neg^CD56^dim^CD57^pos^NKG2C^pos^ NK cells remained significantly elevated in PET versus PRO recipients at 6 months (*P* = 0.03).

**Figure 4:**
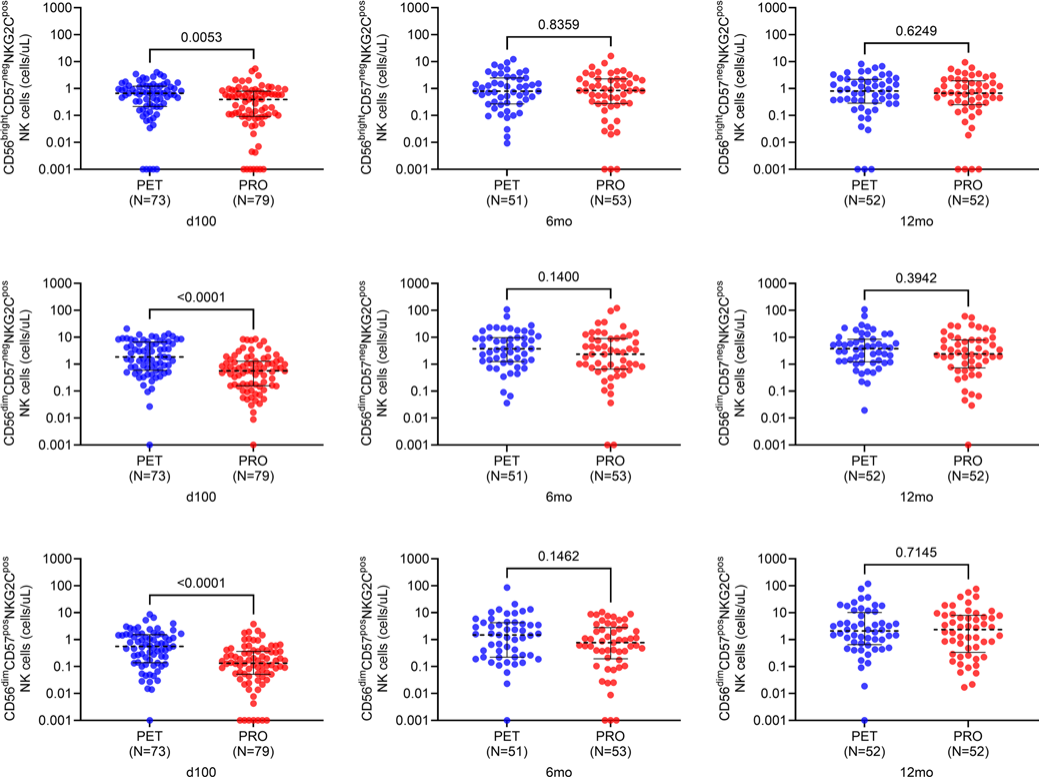
Absolute counts of NK cell subtypes at 100 days, 6 months, and 12 months after transplant. NK cell subsets were categorized based on cell surface level expression of CD56 (i.e., bright vs. dim) and CD57 (i.e., positive vs. negative). Specifically, absolute counts of CD3^neg^CD56^bright^CD57^neg^NKG2C^pos^, CD3^neg^CD56^dim^CD57^neg^NKG2C^pos^, and CD3^neg^CD56^dim^CD57^pos^NKG2C^pos^ NK cells are shown in PET vs. PRO recipients at all 3 time points. For absolute cell counts, 0 values were imputed as a low value (i.e., less than minimum of distribution) for graphing purposes owing to logarithmic scale conversion. Dotted black lines represent median values, and whiskers represent interquartile range. Comparisons were made using 2-sided Wilcoxon’s rank-sum testing at 95% CI.

Next, absolute counts of the above NK cell subsets expressed as cells/mL were calculated for both treatment arms at each time point (Figure 4). Absolute counts of CD3^neg^CD56^dim^CD57^neg^NKG2C^pos^ and CD3^neg^CD56^dim^CD57^pos^NKG2C^pos^ NK cells were significantly higher in the PET versus PRO group at 100 days after transplant (*P* < 0.001 for both, respectively) but not at later time points. Collectively, these data demonstrate differentially higher early expansion of the absolute number and proportion of NKG2C-expressing adaptive NK cells with PET versus PRO.

#### CMV-specific nAbs against epithelial cell entry are increased with PET versus PRO.

**Figure 5:**
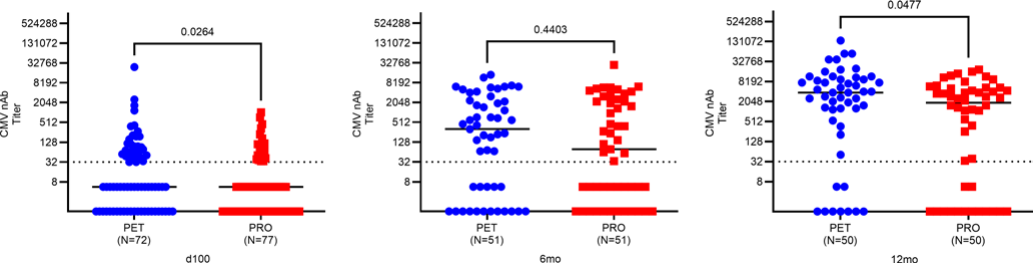
Epithelial cell entry-specific neutralizing antibody titers by treatment arm. Epithelial cell entry-specific neutralizing antibody (nAb) titers at approximately 100 days, 6 months, and 12 months after transplant. Patients were grouped according to treatment arm: PET (blue) vs. PRO (red). Dilution titers were calculated from IC_50_ values for graphing purposes by taking the antilog_2_ of each value. For example, an IC_50_ of 5 corresponds to a CMV nAb dilution titer of 32. Solid black lines represent the median nAb dilution titer for each group. Comparisons were made using 2-sided Wilcoxon’s rank-sum testing at 95% CI.

We compared nAb dilution titers directed against epithelial cell–specific viral entry in PET versus PRO recipients (Figure 5). CMV nAb dilution titers were significantly higher in PET recipients compared with PRO recipients at 100 days and 12 months after transplant (*P* = 0.03 and *P* = 0.05, respectively). Overall, the proportion of patients who developed CMV-specific nAb responses and the relative nAb dilution titer values following transplant increased over time in both study arms.

#### CMV replication in PET recipients is correlated with the development of T cell and nAb immune responses.

**Figure 6:**
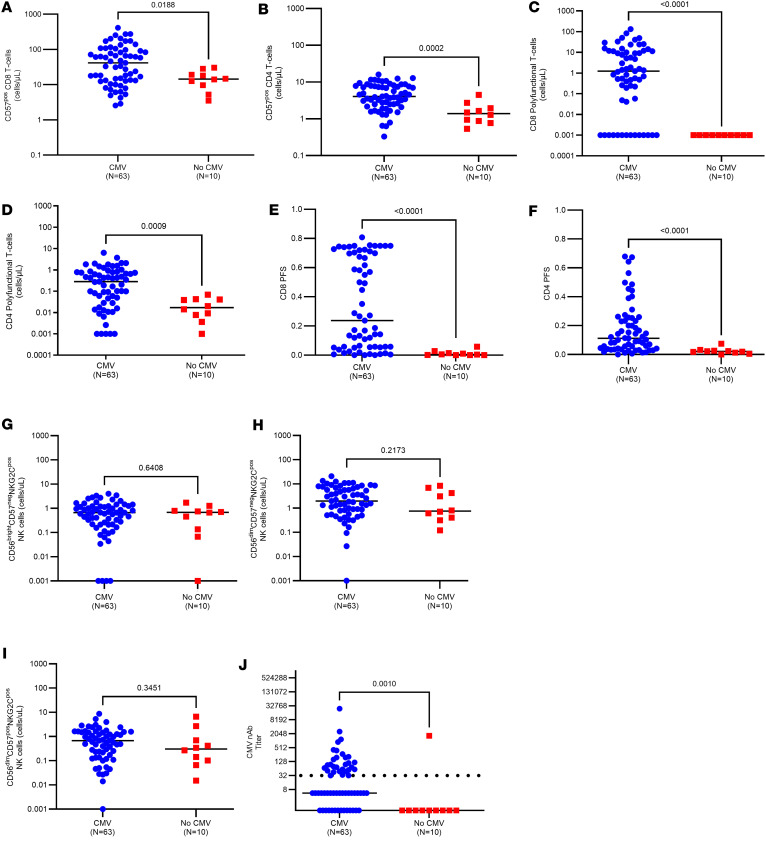
T cell, NK cell, and humoral immune responses in PET recipients with and without preceding CMV DNAemia. Immune parameters at 100 days after transplant in PET recipients (*N* = 73) stratified by preceding detectable CMV DNAemia by qPCR in the first 100 days after transplant. Patients were grouped according to positive (blue) or negative (red) CMV DNAemia in the first 100 days after transplant. Examined immune parameters included terminally differentiated (**A**) CD8^+^ and (**B**) CD4^+^ T cell counts based on the expression of CD57; CMV-specific polyfunctional absolute (**C**) CD8^+^ and (**D**) CD4^+^ T cell counts; COMPASS (**E**) CD8 and (**F**) CD4 polyfunctionality scores (PFSs); absolute counts of (**G**) CD3^neg^CD56^bright^CD57^neg^NKG2C^pos^, (**H**) CD3^neg^CD56^dim^CD57^neg^NKG2C^pos^, (**I**) and CD3^neg^CD56^dim^CD57^pos^NKG2C^pos^ NK cells; and (**J**) CMV epithelial cell entry-specific neutralizing antibody (nAb) dilution titers. Polyfunctional T cell counts were defined as those expressing IFN-γ plus at least 1 additional functional marker. Dilution titers were calculated from IC_50_ values for graphing purposes by taking the antilog_2_ of each value. For example, an IC_50_ of 5 corresponds to a CMV nAb dilution titer of 32. For absolute cell counts, 0 values were imputed as a low value (i.e., less than minimum of distribution) for graphing purposes owing to logarithmic scale conversion. Solid black lines represent values, and whiskers represent interquartile range. Comparisons were made using 2-sided Wilcoxon’s rank-sum testing at 95% CI.

To assess the relationship between CMV replication (as a surrogate for CMV antigen exposure) and the development of CMV-specific immunity in PET recipients, we examined the association of CMV DNAemia (the detection of DNA in samples of plasma, whole blood, and isolated peripheral blood leukocytes or in buffy coat specimens) with the development of each of the examined immune parameters at the end of PET (i.e., 100 days after transplant; Figure 6). Most of the measured immune parameters, including nAb dilution titers, COMPASS scores, antigen-experienced T cells, and CMV-specific polyfunctional T cells, were significantly higher at 100 days among those with preceding CMV viremia, with the exception of the NK cell subsets, which were numerically but not statistically higher. These findings suggest that CMV antigen exposure is the mechanism underlying development of CMV-specific T cell and humoral immunity during PET.

### Association of CMV-specific T cell, NK cell, and nAb responses with postintervention delayed-onset CMV disease

#### CMV-specific polyfunctional T cell and adaptive NK cell immunity is associated with decreased risk of late-onset CMV disease.

**Figure 7:**
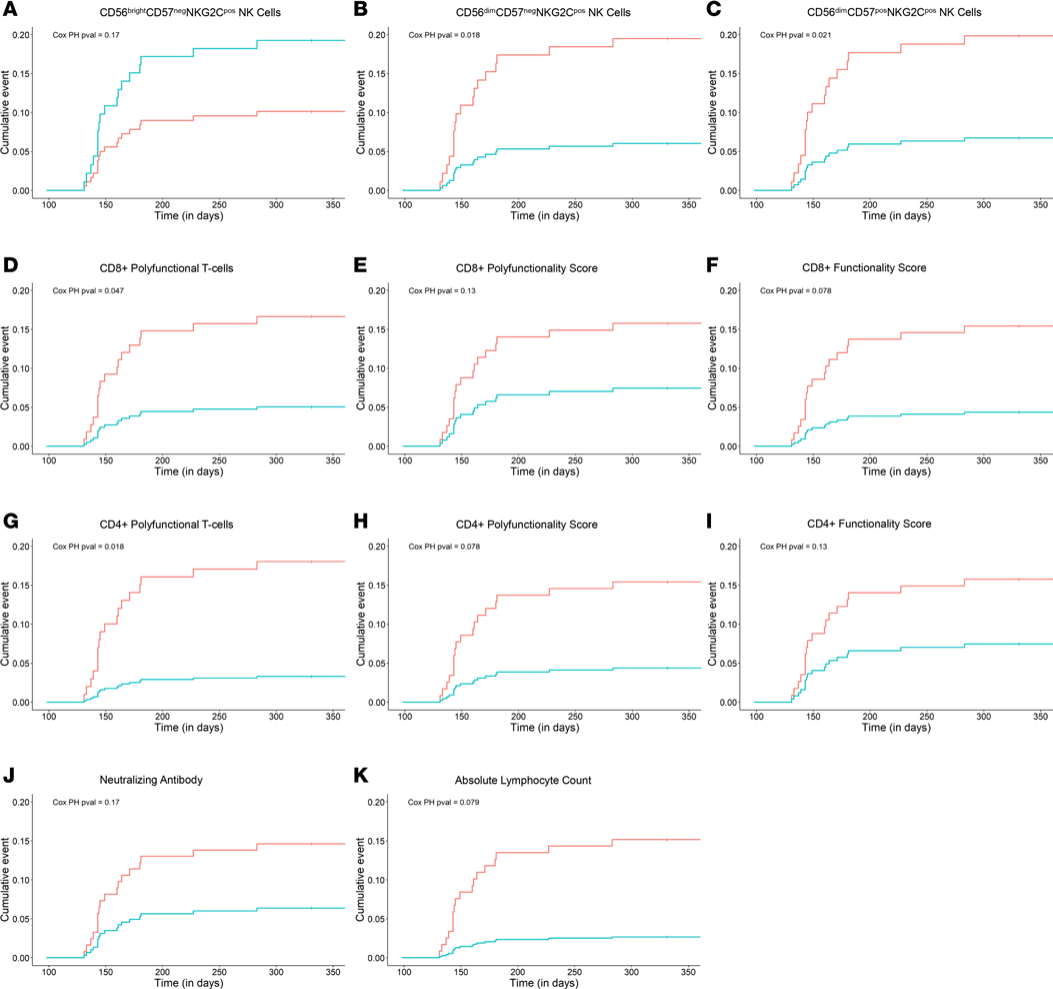
Cumulative incidence of late CMV disease after high-risk, CMV D^+^R^–^ liver transplant stratified by baseline CMV-specific NK cell and T cell immunity. The cumulative incidence of endpoint-adjudicated late CMV disease after high-risk, CMV D^+^R^–^ liver transplant stratified by baseline CMV-specific T cell immunity measured following discontinuation of study intervention after transplant day 100. Time-to-event curves were stratified by the dichotomous threshold cutoffs listed in Table 2 for (**A**) CD3^neg^CD56^bright^CD57^pos^NKG2C^pos^, (**B**) CD3^neg^CD56^dim^CD57^neg^NKG2C^pos^, (**C**) CD3^neg^CD56^dim^CD57^pos^NKG2C^pos^ NK Cells, (**D**) polyfunctional absolute CD8^+^ T cell counts, (**E**) CD8 polyfunctionality scores, (**F**) CD8 functionality scores, (**G**) polyfunctional absolute CD4^+^ T cell counts, (**H**) CD4 polyfunctionality scores, (**I**) CD4 functionality scores, (**J**) neutralizing antibodies, and (**K**) absolute lymphocyte count. Patients with above-threshold levels of immune parameters (blue curves) were compared with patients with below-threshold levels of immune parameters (red curves).

To assess the ability of each immune parameter to predict late-onset CMV disease, we performed univariable Cox proportional hazard (CoxPH) regression and time-to-event analyses at their optimized cutoff thresholds (Supplemental Table 2 and Figure 7). The presence of more than 0 cells/μL CMV-specific polyfunctional CD8^+^ T cells (HR 0.28, 95% CI 0.08–0.98; *P* = 0.047) or more than 0.06 cells/μL CMV-specific polyfunctional CD4^+^ T cells (HR 0.17, 95% CI 0.04–0.73; *P* = 0.02) at 100 days after transplant was associated with a lower risk of late-onset CMV disease. COMPASS scores showed similar associations but were not statistically significant.

Furthermore, the presence of more than 0.54 cells/μL CD3^neg^CD56^dim^CD57^neg^NKG2C^pos^ (HR 0.24, 95% CI 0.09–0.65, *P* = 0.005) or more than 0.32 cells/μL CD3^neg^CD56^dim^CD57^pos^NKG2C^pos^ (HR 0.14, 95% CI 0.03–0.60, *P* = 0.008, respectively) NK cells at 100 days after transplant was associated with a lower risk of CMV disease.

Similarly, as summarized in Figure 7, the proportion of patients who developed late-onset CMV disease was lower in patients with more than 0 cells/μL CMV-specific polyfunctional CD8^+^ T cells or more than 0.06 cells/μL CMV-specific polyfunctional CD4^+^ T cells at 100 days after transplant. In addition, patients with 0.85 cells/μL CD3^neg^CD56^bright^CD57^neg^NKG2C^pos^, more than 0.54 cells/μL CD3^neg^CD56^dim^CD57^neg^NKG2C^pos^, or more than 0.32 cells/μL CD3^neg^CD56^dim^CD57^pos^NKG2C^pos^ NK cells at 100 days after transplant also had a decreased incidence of late-onset CMV disease. These analyses support the concept that selected immune parameters measured at the end of PET or PRO prevention strategies are potential immune correlates for risk of CMV disease in CMV high-risk D^+^R^–^ LTxR.

#### CMV-specific polyfunctional CD4^+^ T cells and antigen-experienced NK cells are protective against late-onset CMV disease after adjusting for nAbs and acute cellular rejection.

To explore whether combinations of cellular and nAb immune parameters after transplant day 100 were predictive of late-onset CMV disease, multivariable CoxPH regression models of T cell and NK cell immune parameters adjusted for nAb dilution titers and acute cellular rejection were constructed based on univariable CoxPH regression results (Table 2). The presence of more than 0.06 cells/μL polyfunctional CD4^+^ T cells at 100 days after transplant was associated with a lower risk of late-onset CMV disease (adjusted HR [aHR] 0.18, 95% CI 0.04–0.82; *P* = 0.03). Furthermore, the presence of more than 0.54 cells/μL CD3^neg^CD56^dim^CD57^neg^NKG2C^pos^ (aHR 0.25, 95% CI 0.09–0.67, *P* = 0.006) or more than 0.32 cells/μL CD3^neg^CD56^dim^CD57^pos^NKG2C^pos^ (aHR 0.15, 95% CI 0.03–0.66, *P* = 0.01) NK cells at 100 days after transplant was also associated with a lower risk of late-onset CMV disease. We corrected for multiple comparisons using Benjamini-Hochberg adjustment, and the results of this analysis are shown in Supplemental Table 3. Following adjustment, the strongest associations remained with polyfunctional CD4^+^ T cell counts (*P* = 0.10), CD3^neg^CD56^dim^CD57^neg^NKG2C^pos^ (*P* = 0.05), and CD3^neg^CD56^dim^CD57^pos^NKG2C^pos^ (*P* = 0.05) NK cells.

**Figure 8:**
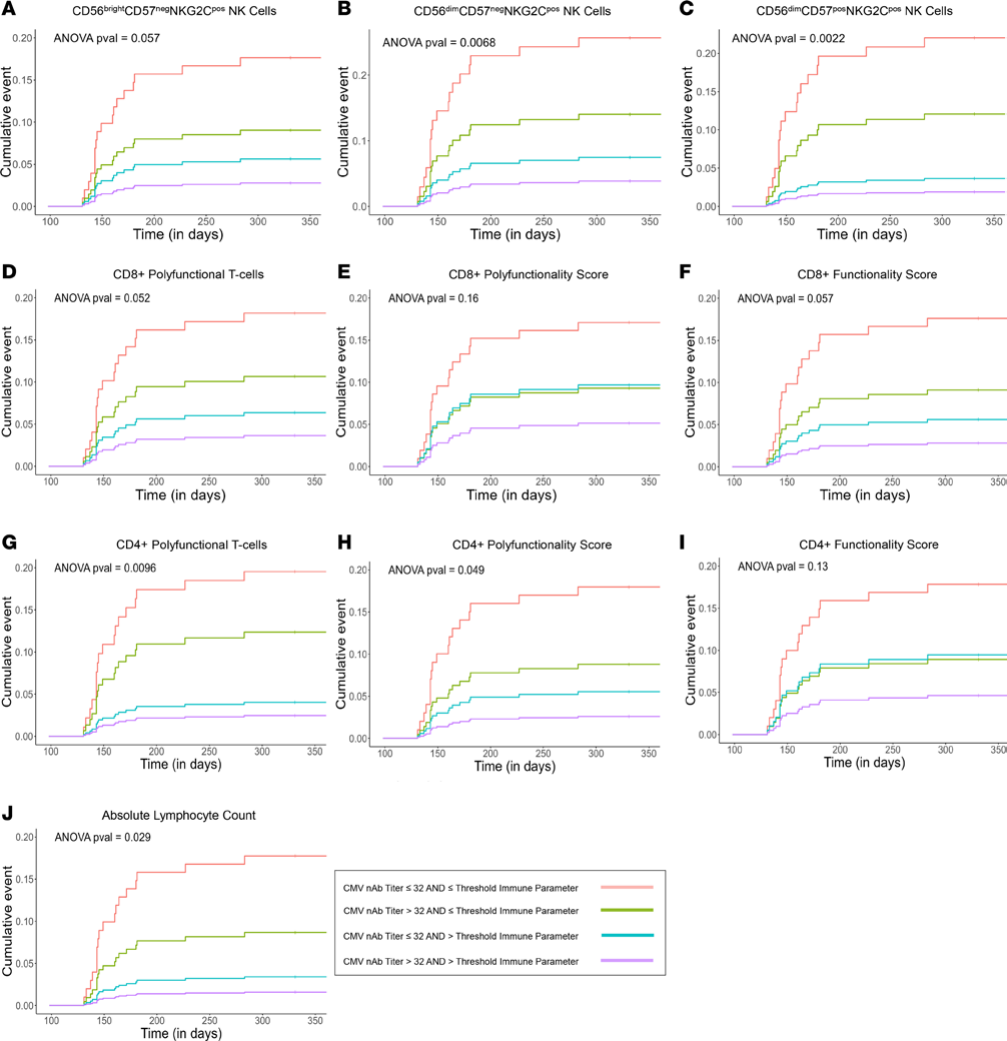
Cumulative incidence of late CMV disease after high-risk, CMV D^+^R^–^ liver transplant stratified by baseline CMV-specific NK cell or T cell immunity with neutralizing antibody titers. The cumulative incidence of endpoint-adjudicated delayed-onset CMV disease following high-risk, CMV D^+^R^–^ liver transplant according to combined cellular and humoral immune parameters measured following discontinuation of study intervention after transplant day 100. Time-to-event curves were stratified by posttransplant day 100 immunity above (purple and teal curves) or below (green and red curves) the dichotomous thresholds listed in Table 2 for (**A**) CD3^neg^CD56^bright^CD57^neg^NKG2C^pos^, (**B**) CD3^neg^CD56^dim^CD57^neg^NKG2C^pos^, (**C**) CD3^neg^CD56^dim^CD57^pos^NKG2C^pos^ NK cells, (**D**) polyfunctional absolute CD8^+^ T cell counts, (**E**) CD8 polyfunctionality scores, (**F**) CD8 functionality scores, (**G**) polyfunctional absolute CD4^+^ T cell counts, (**H**) CD4 polyfunctionality scores, (**I**) CD4 functionality scores, and (**J**) absolute lymphocyte count combined with neutralizing antibody dilution titers >32 (green and purple curves) or ≤32 (red and teal curves), which is equivalent to a log_2_ neutralizing antibody (nAb) dilution titer (i.e., IC_50_) of 5.

The cumulative incidence of CMV disease after day 100 stratified by each T cell and NK cell immune parameter (above or below each dichotomous threshold), in combination with log_2_ nAb dilution titers (i.e., IC_50_) >5 or ≤5 is shown in Figure 8. Patients with below-threshold levels of all NK cell or T cell immune parameters and log_2_ nAb dilution titers ≤5 had the highest incidence of late-onset CMV disease. The largest increased incidence of CMV disease was observed with ≤0.32 cells/μL of CD3^neg^CD56^dim^CD57^pos^NKG2C^pos^ NK cells or ≤0.06 cells/μL of CMV-specific polyfunctional CD4^+^ T cells combined with nAb dilution titers ≤32 at 100 days after transplant, implicating these immune parameters with protection against late-onset CMV disease.

Alternative iterations of the above analyses were performed in which patients with the highest incidence of late CMV disease (i.e., patients with below-threshold levels of any of the evaluated NK cell or T cell immune parameters combined with nAb dilution titers ≤32) were considered the “reference group”; patients with above-threshold levels of any of the evaluated NK cell or T cell immune parameters and/or nAb dilution titers above 32 were combined into a single “comparator group” (Supplemental Figure 5). Patients with nAb dilution titers of more than 32 with or without either more than 0 cells/μL of CMV-specific polyfunctional CD8^+^ T cells (*P* = 0.04) or more than 0.06 cells/μL of CMV-specific polyfunctional CD4^+^ T cells (*P* = 0.03) at 100 days after transplant were at a statistically lower risk of late-onset CMV disease compared with the highest-risk patients. Similarly, patients with nAb dilution titers of more than 32 with or without 0.85 cells/μL CD3^neg^CD56^bright^CD57^neg^NKG2C^pos^ (*P* = 0.03), 0.54 cells/μL of CD3^neg^CD56^dim^CD57^neg^NKG2C^pos^ (*P* = 0.005), and 0.32 cells/μL CD3^neg^CD56^dim^CD57^pos^NKG2C^pos^ (*P* = 0.007) NK cells were at a lower risk of late CMV disease compared with the highest-risk patients. This alternative analytical approach corroborated the finding that the highest-risk group for late-onset CMV disease were patients with below-threshold levels of any of the evaluated NK cell or T cell immune parameters combined with nAb dilution titers ≤32.

#### Principal component analysis of T cell, NK cell, and nAb immunity at 100 days after transplant.

**Figure 9:**
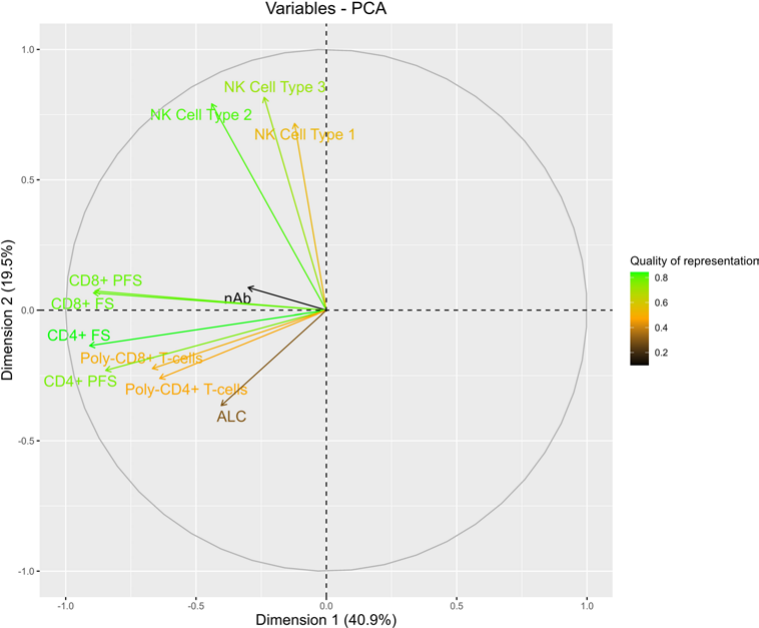
Variable correlation plots of principal component analysis results. Variable correlation plots of relationships between all examined immune parameters based on principal component analysis (PCA) results. Positively correlated immune parameters are grouped together, whereas negatively correlated immune parameters appear on opposite sides of the plot origin. The quality immune parameter representation in the PCA is displayed according to the distance of each immune parameter vector and the origin the square cosine (i.e., cos^2^) of each immune parameter where a high cos^2^ (i.e., green vector) indicates good representation and a low cos^2^ (i.e., black vector) indicates poor representation.

Given the high dimensionality of the data and potential correlations between measured parameters at 100 days after transplant, principal component (PC) analysis was used. Eleven PCs were evaluated, and individual loadings for each are shown in Supplemental Table 4. Scree plots were used to compare the proportion of variation accounted for by each PC (Supplemental Figure 6). PC1 and PC2 accounted for 60.4% of the total variance in the data. Correlation plots were created to visualize the quality of representation and correlations in the data according to these PCs (Figure 9). Overall, all NK cell parameters were highly correlated, as were polyfunctional T cell counts; however, NK cell and polyfunctional T cell counts appeared negatively correlated with each other. Interestingly, the two variables with the highest quality of representation in the PC analysis (PCA) included CD3^neg^CD56^dim^CD57^neg^NKG2C^pos^ NK cells and CMV-specific CD4 FSs. These findings show that CMV-specific polyfunctional T cell and adaptive NK cell immunity continue to be critically associated with protection against late-onset CMV disease, even when considering the high dimensionality and correlations in the data.

#### Performance characteristics of CMV-specific T cell, NK cell, and nAb responses to predict delayed-onset CMV disease.

We evaluated the performance characteristics of each immune parameter, dichotomized by their respective optimized threshold, to predict CMV disease by 1 year after transplant after adjusting for nAbs and acute cellular rejection (Table 3). Overall, CD3^neg^CD56^dim^CD57^pos^NKG2C^pos^ NK cells had the most optimal performance, with a sensitivity of 0.889, specificity of 0.496, positive predictive value (PPV) of 0.195, and negative predictive value (NPV) of 0.970. The performance characteristics of the PCA had a sensitivity of 0.822, specificity of 0.574, PPV of 0.209, and NPV of 0.959. Thus, the PCA was similar in the ability to predict late-onset CMV disease to each individual parameter evaluated independently after adjustment for nAb dilution titers and acute cellular rejection.

## Discussion

CMV high-risk D^+^R^–^ LTxRs randomized to PET for 100 days after transplant had significantly higher CMV-specific IFN-γ–expressing polyfunctional T cells, NK cell subsets, and nAbs compared with PRO recipients. The association between preceding CMV viremia and subsequent development of CMV-specific T cell and nAb responses implicates greater CMV antigen exposure during viral replication in PET compared with PRO as the underlying mechanism for the observed higher immune responses in the PET group. Finally, in multivariable models, increased CMV-specific polyfunctional CD4^+^ T cells, CD3^neg^CD56^dim^CD57^neg^NKG2C^pos^ cells, and CD3^neg^CD56^dim^CD57^pos^NKG2C^pos^ NK cells were each independently associated with protection against the clinically relevant outcome of late-onset CMV disease. Collectively, these findings suggest that PET, through controlled viral antigen exposure, better facilitates development of CMV-specific immune responses (compared with PRO) and that these immune responses mediate CMV protective immunity against CMV disease among high-risk D^+^R^–^ SOT recipients.

In our study, CMV-specific polyfunctional T cell responses were increased in PET versus PRO recipients. These findings are consistent with previous studies of immune function after SOT and HSCT (34, 37). The observed longer-lasting (at 1 year) increase in CMV-specific polyfunctional CD8^+^ T cell responses with PET also aligns with small observational studies of CMV-specific T cell immunity in SOT recipients (38, 39). In contrast, although CMV-specific polyfunctional CD4^+^ T cell responses were higher at 100 days after transplant in the PET group, this was not sustained at later time points. It is possible that CMV-specific CD4^+^ T cells undergo a differentiation process that causes them to be less responsive to CMV antigen stimulation over time (40). This could explain the observed differences in longitudinally measured CMV-specific CD4^+^ T cell immunity between treatment groups. CMV-specific polyfunctional T cell immunity was assessed by in vitro stimulation with an overlapping peptide pool of CMV pp65. However, other CMV antigens are expressed during viral replication, including antigens not measured in the current study (11, 34, 41). No longitudinal or qualitative differences were observed in COMPASS scores, which are calculated independently of the number of circulating T cells, following stimulation with our positive control superantigen (SEB). Thus, PET likely leads to differential alterations in CMV-specific functional responses rather than alterations in global immune function from immunosuppression or valganciclovir-related lymphotoxicity between the PET and PRO groups (42).

The strong correlation between CMV DNAemia and higher CMV-specific polyfunctional T cell immunity in the PET group supports the hypothesis that CMV antigen exposure drives this expansion. The hypothesis is further supported by mouse studies showing rapid expansion of murine CMV–specific CD8^+^ T cells following primary murine CMV infection (43, 44) and by T cell receptor studies of T cell clonal expansion following primary CMV infection after SOT (45, 46). A specific threshold of CMV viremia with PET that predicted development of a T cell response was not identified in this study but is important for future research. Although CMV DNAemia was not routinely assessed in the PRO group, the incidence has consistently been reported as less than 5%–10% in prior randomized trials (47), compared with the observed approximately 80% incidence with PET in the current study. In addition, although the duration (total days) of valganciclovir exposure was longer with PRO compared with PET in the CAPSIL trial, the total drug exposure (mg/person) between groups was not markedly different (14). This is likely explained by the treatment dosing used in the PET group (twice daily) versus the PRO group once daily prophylaxis dosing. Thus, despite the relatively similar total drug exposure between groups, there were substantially higher CMV-specific immune responses with PET. This further implicates greater antigen exposure with PET as the key driver of enhanced CMV-specific immunity in the PET versus PRO groups.

NKG2C-expressing NK cells have been shown to be elevated in previously CMV-infected individuals; therefore, it can be said that these cells represent an “adaptive” or “memory-like” cell population (48–52). In addition, coexpression of CD57 by these NKG2C-expressing NK cells is proposed to represent a more educated or “antigen-experienced” subset of these cells (36). In our study, PET recipients had increased absolute counts and proportions of CD3^neg^CD56^dim^CD57^neg^NKG2C^pos^ and CD3^neg^CD56^dim^CD57^pos^NKG2C^pos^ NK cells at the end of study intervention. Interestingly, there were no significant differences in NK cells in PET recipients with or without preceding CMV viremia, suggesting that other measures of CMV antigen exposure (e.g., local CMV replication in the allograft) might be important for expansion of adaptive NK cell responses. The differences in adaptive NK cells between treatment arms appeared to diminish over time, possibly reflecting rapidly increased CMV antigen exposure after day 100 in the PRO group.

A greater degree of pathogen-specific T cell polyfunctionality has been correlated with improved immune protection and nonprogression of other infections (53–55). Polyfunctional T cell responses, particularly those that include IFN-γ, have been associated with protection against CMV infection/disease in SOTr in prior studies (15, 17). Furthermore, some in vitro studies have shown distinct molecular patterns between monofunctional and polyfunctional T cells at the transcriptome level, which may contribute to the enhanced immune protection offered by the latter (56). We observed a decreased risk of late CMV disease in association with several polyfunctional T cell parameters. These findings are consistent with data from small cohort studies linking polyfunctional CMV-specific T cell immunity with reduced risk of subsequent CMV disease following D^+^R^–^ lung (17) and liver transplantation (57). In our study, CMV-specific polyfunctional CD4^+^ T cells were independently associated with protection against CMV disease and decreased (i.e., below-threshold) levels were predictive of subsequent CMV disease. These findings are consistent with other smaller studies that have showed a possible role for CD4^+^ T cell immune protection against CMV after SOT (15, 17, 58–60).

Although there are limited data describing the protective capacity of NK cells against CMV in high-risk D^+^R^–^ SOT recipients (23, 61), statistically significant reductions in the cumulative incidence of late-onset CMV disease were observed with increased levels of multiple NK cell subtypes at 100 days after transplant. Our findings parallel a recent study on the protective role of NK cells against late-onset CMV infection in HCT recipients who received letermovir prophylaxis (62). Furthermore, the potential importance of nAbs against CMV pentameric complex in protection against CMV infection is only beginning to be explored (63). The findings of a decreased CMV disease incidence in patients with higher nAb titers at 100 days after transplant contrasts with a recent study in which CMV D^+^R^–^ kidney transplant recipients who received PRO and underwent T cell–depleting induction showed no protective association for nAbs against CMV infection/disease (64). Our findings are more consistent with the decreased incidence of CMV disease observed among CMV D^+^R^–^ kidney transplant recipients randomized to receive monoclonal antibody against pentameric complex (32, 65).

The use of well-characterized patient samples from a clinical trial allowed us to explore the relationship between multiple immune parameters with the clinically relevant outcome of adjudicated CMV disease. After adjusting for nAb dilution titers and acute cellular rejection, CMV-specific polyfunctional CD4^+^ T cells, CD3^neg^CD56^dim^CD57^neg^NKG2C^pos^, and CD3^neg^CD56^dim^CD57^pos^NKG2C^pos^ NK cells remained independently associated with a decreased risk of late-onset CMV disease. Low levels of the combination of CMV-specific nAb and T cell immunity were associated with a increased incidence of late-onset of CMV disease. In addition, low-level nAb and either CMV-specific polyfunctional T cell or adaptive NK cell immune responses were highly predictive (i.e., high NPV) of subsequent CMV disease and, for most immune parameters, the predictive ability was improved in combination with nAb titers. These findings may be attributed to an interaction between CMV-specific humoral and cellular immunity via antibody-dependent cellular cytotoxicity (66, 67). Findings from our PC analyses provide clues to the relative importance of each immune parameter for protection against CMV, with CMV-specific polyfunctional T cell and NK cell responses having the greatest representation. Collectively, our findings suggest that T cell, NK cell, and nAb immunity may all contribute to protection against CMV disease in the D^+^R^–^ primary infection SOT setting and that there may be value in combined assessment of multiple immune parameters.

Our study opens avenues for future investigation into T cell, NK cell, and humoral immune responses in high-risk CMV D^+^R^–^ SOTr and their influence on the risk for CMV disease. For example, it remains unclear if differences in CMV-specific immune responses with PET versus PRO could be attributed to valganciclovir-related toxicity (68–70). Although ganciclovir decreases lymphocyte proliferation, polyfunctional CMV-specific T cell immunity has previously been shown to be largely unaffected in vitro (71–73). Because of this and the pharmacokinetic properties of valganciclovir, global nonspecific valganciclovir-associated immune cell toxicity with PRO is less likely to explain the differences in immune parameters between groups. However, this should be assessed in future studies. In addition, assessment of CMV-specific polyfunctional T cell immunity to a broader range of CMV antigens (e.g., IE1, IE2) is important to better characterize the full breadth and quality of CMV immune responses and their relationship with CMV disease.

The independent association of CMV-specific polyfunctional CD4^+^ T cells with protection against CMV disease in a large cohort of patients within the context of a randomized trial is an important finding of this study and identifies a potential target for future immune-based interventions. Furthermore, enhanced CMV-specific immunity in PET recipients up to 12 months after transplant (~9 months after discontinuation of the primary intervention) has important clinical implications. The finding is particularly relevant in SOT recipients who require lifelong immunosuppression, with its associated risk for long-term CMV reactivation and association with worse graft and patient survival (74–77). In post hoc analyses of the CAPSIL trial, there was improved long-term survival with PET compared with PRO, suggesting that improved CMV-specific immunity, by better long-term control of subclinical CMV replication, may be associated with improved overall SOT outcomes (14).

The study has strengths. First, samples were derived from a large and well-characterized patient population in the context of a protocolized multicenter randomized controlled trial that included longitudinal samples collected up to 12 months after transplant in a high-risk CMV D^+^R^–^ population (14). We were able to assess the predictive capability of each immune parameter for a clinically relevant endpoint of CMV disease that was assessed by an endpoint committee. All immunologic analyses were performed at a central lab by personnel blinded to clinical status (e.g., study arm, CMV disease). We acknowledge potential study limitations. Even though this is one of the largest studies to assess the association of multiple CMV immune parameters with CMV disease risk, the total number of disease events was small and precluded the ability to adjust for multiple comparisons. Thus, the putative immune correlates identified here should be confirmed and validated in future studies. Not all randomized participants had all time points available for immune function testing owing to poor cell viability and/or low cell counts, which theoretically could have been due to freezing and thawing of these clinical samples. However, blood processing and freezing was performed at a single central laboratory by blinded personnel and cell viability was similar between study arms. In addition, the characteristics and outcomes of included versus excluded patients from the current study were similar. We assessed CMV-specific polyfunctional T cell immune responses only to pp65; however, responses to other immunodominant antigens may also be important (17). Additionally, it is known that the T cell response encompasses a broad array of CMV antigens (41), which may also account for differences in absolute lymphocyte counts seen in the original trial (14). Although our study is one of the few studies to integrate T cell and NK cell immunity with nAb responses, there may be other specific antibody function(s) that contribute to protective immunity in this setting of CMV D^+^R^–^ SOTr, such as antibody-dependent cellular cytotoxicity, antibody-dependent cellular phagocytosis, or complement-dependent cytotoxicity (78). Finally, determination of whether the higher CMV-specific immune responses seen with PET compared with PRO persisted beyond 1 year was not assessed.

In conclusion, PET is associated with significantly higher and longer lasting CMV-specific polyfunctional T cell, adaptive NK cell, and nAb responses in high-risk CMV D^+^R^–^ LTxRs compared with PRO, and greater CMV antigen exposure during CMV replication during PET is likely important for the development of these CMV-specific immune responses. CMV-specific polyfunctional CD4^+^ T cells, CD3^neg^CD56^dim^CD57^neg^NKG2C^pos^ cells, and CD3^neg^CD56^dim^CD57^pos^NKG2C^pos^ NK cells were each independently associated with protection against CMV disease, paving the way for assessment of these parameters as immune correlates in future studies. Collectively, these findings suggest that controlled antigen exposure during PET versus PRO better facilitates durable CMV protective immunity rather than the approach of complete viral suppression (PRO). The specific immune correlates of CMV protective immunity and relative contributions of T cell, NK cell, and nAb immunity require further study.

## Methods

### Sex as a biological variable.

The CAPSIL trial (NCT01552369) included 62 female and 143 male participants.

### Study population and design.

The CAPSIL trial included 205 CMV D^+^R^–^ LTxRs (100 PET, 105 PRO). Baseline characteristics and randomization procedures were previously reported (14). All patients with samples tested by both flow cytometry and nAb assays were included in comparative analyses of CMV-specific immunity between study arms because the primary outcome was the development of CMV-specific immune responses. For analyses of the association of CMV-specific immunity at day 100 and risk of late CMV disease, participants who developed CMV disease before day 100 after transplant were excluded. All immune analyses were performed by personnel who were blinded to treatment assignment and clinical outcomes to minimize bias.

### Intracellular cytokine staining and flow cytometry.

PBMCs collected at approximately 100 days, 6 months, and 12 months after transplant were tested using a 17-color intracellular cytokine staining assay modified from previously published protocols (79, 80). Cells were stained using the following fluorescent antibodies: CD3 BUV395, CD8 BUV805, CD4 BUV496, IL-2 PE, IFN-γ V450, CD154 APC, CD45RA BUV737, and CD56 BV650 (all BD Biosciences); CD14 BV605, CCR7 BV785, PD-1 PE-Dazzle594, IL-4 PerCPCy5.5, and Perforin PECy7 (all from Biolegend); blue fixable viability dye and TNF-α FITC (both from Thermo Fisher Scientific); CD57 APC-Vio770 (Miltenyi); and NKG2C AlexaFluor700 (R&D Systems). Catalog and clone numbers are included in Supplemental Table 5.

Cell acquisition (at 100,000–400,000 events) was performed using a Symphony flow cytometer (BD Biosciences) within 24 hours of staining. All antibodies were titrated for optimum performance, and appropriate single-color compensation and fluorescence minus-one controls were run. Data were analyzed using FlowJo software (version 9.9.6), and the gating strategy is shown (Supplemental Figure 2).

### Antigen-experienced and CMV-specific polyfunctional T cells.

Antigen-experienced T cells were defined as unstimulated CD8^+^ or CD4^+^ T cells that coexpressed CD57. Functional CD8^+^ and CD4^+^ T cell immune responses were measured in response to stimulation with CMV pp65 peptide library or SEB. “Polyfunctional” CMV-specific T cell subsets were defined as those that expressed “IFN-γ plus at least 1 additional measured functional marker” (i.e., TNFA, IL2, CD154, or PRF1). Immune responses were background subtracted using DMSO as negative control responses (14, 79, 80). Positive responses were defined as T cell frequencies greater than 0.05% above background and at least 3-fold greater than DMSO response in the same cell population (14). Responses that did not meet these criteria were set to 0 for statistical purposes. Clinical absolute lymphocyte counts at each time point were used to transform percentage of parent data to calculate absolute cell counts. Simplified Presentation of Incredibly Complex Evaluations (SPICE) version 6.1 was used to summarize polyfunctional T cell phenotypes for positive responses only (81). For SPICE, CD154 and IL-4 were removed from calculation of polyfunctional CD8^+^ T cell while IL4 responses were removed from calculation of polyfunctional CD4^+^ T cell responses given low expression in these cell compartments, respectively (82, 83).

### COMPASS.

COMPASS was also used to assess T cell polyfunctionality (34). This approach has the advantage of identifying possible immune correlates of protection that would have otherwise been missed by more conventional measurements of T cell immunity (33). PFSs and FSs were generated using COMPASS to summarize functional T cell responses. PFS differs from FS by weighing T cell subsets by the degree of their polyfunctionality (i.e., cell subsets that respond to antigen with a greater number of markers receive larger weight) (33, 34). Similar to SPICE, CD154 and IL-4 were removed from calculation of polyfunctional CD8^+^ T cell responses, whereas IL-4 as removed from calculation of polyfunctional CD4^+^ T cell responses.

### Adaptive NK cell subsets.

NK cells were defined by the combined absence of CD3 and by the level of expression of CD56 (i.e., CD56^bright^ or CD56^dim^). We focused on antigen-experienced NKG2C-expressing NK cell subsets based on the absence or presence of CD57. Three NK cell phenotypic populations were defined based on the combination of these markers: CD3^neg^CD56^bright^CD57^neg^NKG2C^pos^, CD3^neg^CD56^dim^CD57^neg^NKG2C^pos^, and CD3^neg^CD56^dim^CD57^pos^NKG2C^pos^ NK cells. Similar to T cells, clinical absolute lymphocyte counts were used to transform flow cytometry data to absolute NK cell counts.

### CMV-specific nAbs.

CMV-specific nAbs activity directed against epithelial cell–specific viral entry was measured using an assay adapted from previously published protocols (84, 85). Details of this assay have previously been described (79, 86).

### Statistics.

Fisher’s exact or χ^2^ tests were used to assess differences in demographics between patients in the original trial and the current study. Absolute polyfunctional T cell counts, COMPASS scores, NK cells, and nAb titers were compared between PET and PRO groups using 2-sided Wilcoxon’s rank-sum tests at the 95% CI. CD4^+^ and CD8^+^ T cell responses were analyzed separately, as both have been implicated in protection against CMV infection/disease (37, 87, 88). To assess whether CMV infection facilitates the development of CMV-specific immunity following PET, immune parameters at day 100 were compared between PET recipients with or without preceding CMV viremia. The ability of each immune parameter to predict late-onset CMV disease (regardless of PRO or PET treatment assignment) was estimated using CoxPH regression with respect to immunity measured after transplant day 100. Prior to the construction of CoxPH models, immune parameters were divided into multiple quantiles/percentiles to optimize the predictive ability of late CMV disease for each immune parameter. Multiple dichotomous cutoff thresholds were tested by dividing immune parameters according to concordance indices (i.e., C-indices, data not shown). For nAbs, a cutoff titer of 32 (which is equivalent to an IC_50_ of 5) was selected based on previously published studies (14, 86). Following identification of optimal cutoff thresholds, multivariable CoxPH regression models were created, adjusting for nAb titers and acute graft rejection. Statistical correction for multiple testing to decrease the false discovery rate was performed using the Benjamini-Hochberg procedure. Given the high dimensionality of immune data and possibility for correlation between immune parameters, PCA was used to deconvolute immune data into separate linearly uncorrelated PCs. Scree plots were generated to describe the proportion of variation, and correlation plots were created to visualize the quality of representation/correlation between variables within PCs. Performance characteristics were calculated for immune parameters and PCs to predict endpoint committee–adjudicated CMV disease up to 1 year after transplant. Cumulative incidence of CMV disease from 100 days to 1 year after transplant was determined with death as a competing risk in the in the “cmprsk” package in the R statistical computing environment, version 3.5.0 (89). *P* values of less than 0.05 were considered significant.

### Study approval.

The CAPSIL trial was approved by the appropriate IRBs at the University of Washington, UCLA Medical Center, Mayo Clinic, Emory University, and the University of Pittsburgh. All participants provided informed consent, and this study was approved by the University of Washington IRB.

### Data availability.

Values for all data points in graphs are reported in the Supporting Data Values file. Sample data are available from the corresponding author upon request. Requests for deidentified stored samples can be made to one of the co–senior authors (APL), with the execution of a materials transfer agreement.

## Author contributions

DZ, SD, DMK, and APL had full access to the data and take responsibility for its integrity and accuracy. NS, DJW, GML, BE, MB, DMK, RRR, AKM, and APL were responsible for the study concept and design. TSA assisted with performing assays and with data interpretation. DZ and SD were responsible for statistical analyses. NS, MB, and APL were responsible for obtaining funding. All authors were involved in drafting and critical review of the manuscript.

## Supplementary Material

Supplemental data

Supporting data values

## Figures and Tables

**Table 1:**
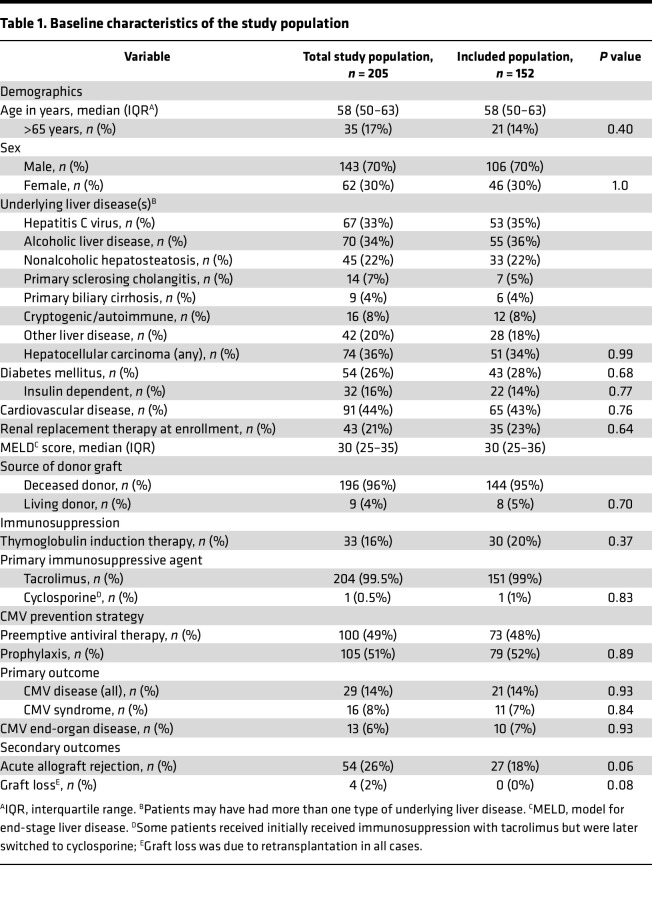
Baseline characteristics of the study population

**Table 2:**
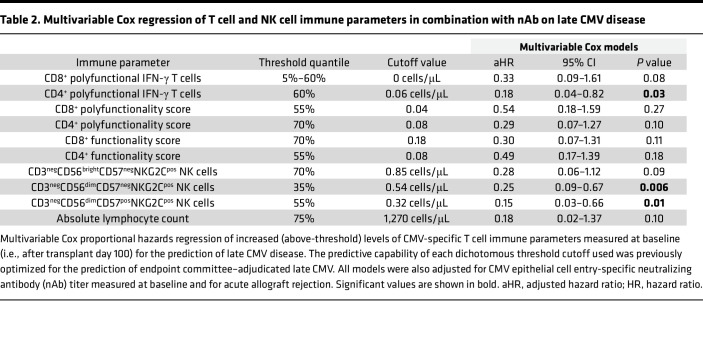
Multivariable Cox regression of T cell and NK cell immune parameters in combination with nAb on late CMV disease

**Table 3:**
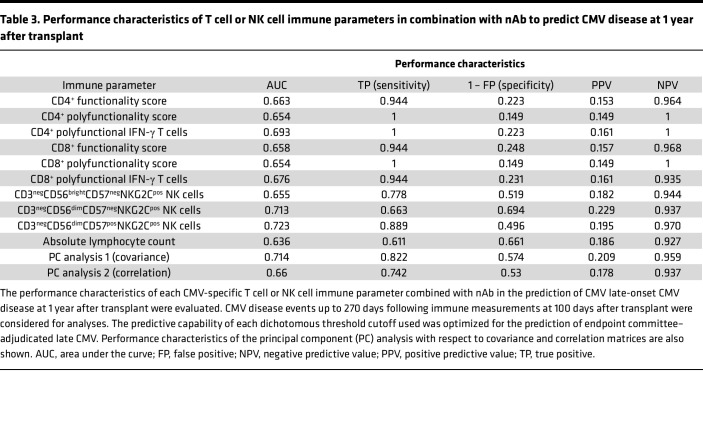
Performance characteristics of T cell or NK cell immune parameters in combination with nAb to predict CMV disease at 1 year after transplant

## Version 1 08/05/2024

In-Press Preview

## Version 2 09/24/2024

Electronic publication
