# Supplementary material for: Cytomegalovirus immunity in high-risk liver transplant recipients following preemptive antiviral therapy versus prophylaxis
Source: JCI Insight. 2024 Sep 24;9(18):e180115. doi: 10.1172/jci.insight.180115 (PMC11457861; doi:10.1172/jci.insight.180115)
Supplement: Supplemental data [file jciinsight-9-180115-s227.pdf]

Variable		Original PET (n=100)	Current PET (n=73)	P-value	Original PRO (n=105)	Current PRO (n=79)	P-value
**Demographics**							
Age	Median (IQR[A])	57 (50-63)	57 (50-61.8)		58 (51-63)	58 (53-63)	
	>65 years	19 (19)	12 (16)	0.66	16 (15)	9 (11)	0.45
Gender	Male	65 (65)	49 (67)		78 (74)	57 (72)	
	Female	35 (35)	24 (33)	0.77	27 (26)	22 (28)	0.75
Underlying liver disease(s)[B]:							
	Hepatitis C virus	30 (30)	22 (30)		37 (35)	31 (39)	
	Alcoholic liver disease	32 (32)	23 (32)		38 (36)	32 (41)	
	Non-alcoholic hepatosteatosis	19 (19)	16 (22)		26 (25)	17 (22)	
	Primary sclerosing cholangitis	9 (9)	6 (8)		5 (5)	1 (1)	
	Primary biliary cirrhosis	6 (6)	3 (4)		3 (3)	3 (4)	
	Cryptogenic/autoimmune	7 (7)	4 (5)		9 (9)	8 (10)	
	Other liver disease	20 (20)	14 (19)		22 (21)	14 (18)	
	Hepatocellular carcinoma (any)	37 (37)	25 (34)	0.99	37 (35)	26 (33)	0.90
Diabetes mellitus		23 (23)	17 (23)	0.96	31 (30)	26 (33)	0.62
	Insulin dependent	16 (16)	11 (15)	0.87	16 (15)	11 (14)	0.80
Cardiovascular disease		43 (43)	28 (38)	0.54	46 (44)	39 (49)	0.45
Renal replacement therapy at enrollment		19 (19)	16 (22)	0.64	24 (23)	19 (24)	0.85
MELD[C] score, median (IQR)		30 (25-35)	30 (25-36)		30 (25-35)	30 (25-36)	
Source of donor graft:							
	Deceased donation	94 (94)	68 (93)		102 (97)	76 (96)	
	Living donation	6 (6)	5 (7)	0.82	3 (3)	3 (4)	0.72
**Immunosuppression**							
Thymoglobulin induction		15 (15)	14 (19)	0.47	18 (17)	16 (20)	0.59
Primary immunosuppressive agent:							
	Tacrolimus	99 (99)	72 (99)		105 (100)	79 (100)	
	Cyclosporine[D]	1 (1)	1 (1)	0.82	0	0	1.0
**Primary Outcome**							
CMV Infection		9 (9)	6 (8)	0.96	20 (19)	15 (19)	1.0
Type of CMV Infection:							
	Syndrome	5 (5)	3 (4)		11 (11)	8 (10)	
	End organ	4 (4)	3 (4)		9 (9)	7 (9)	

[A]IQR=interquartile range; [B]Patients may have had more than one type of underlying liver disease; [C]MELD=Model for End-stage Liver Disease; [D]Some patients initially received immunosuppression with tacrolimus but were later switched to cyclosporine

Supplemental Table 1. – Baseline characteristics of the study population by treatment arm.

			Univariable Cox Models		
**Immune Parameter**	**Threshold Quantile**	**Cutoff Value**	**HR**	**95% CI**	**p-value**
CD8 Polyfunctional T-cells	5%-60%	0 cells/uL	0.28	0.08-0.98	**0.05**
CD8 Polyfunctionality Score	55%	0.04	0.45	0.16-1.27	0.13
CD8 Functionality Score	70%	0.18	0.27	0.06-1.16	0.08
CD4 Polyfunctional T-cells	60%	0.06 cells/uL	0.17	0.04-0.73	**0.02**
CD4 Polyfunctionality Score	70%	0.08	0.27	0.06-1.16	0.08
CD4 Functionality Score	55%	0.08	0.45	0.16-1.27	0.13
$CD3^{neg}CD56^{bright}CD57^{neg}NKG2C^{pos}$ NK Cells	70%	0.85 cells/uL	0.27	0.06-1.17	0.08
$CD3^{neg}CD56^{dim}CD57^{neg}NKG2C^{pos}$ NK Cells	35%	0.54 cells/uL	0.24	0.09-0.65	**0.005**
$CD3^{neg}CD56^{dim}CD57^{pos}NKG2C^{pos}$ NK Cells	55%	0.32 cells/uL	0.14	0.03-0.60	**0.008**
Absolute Lymphocyte Count	75%	1210 cells/uL	0.16	0.02-1.24	0.08
CMV Neutralizing Antibody Titer[A]	-	32	0.42	0.12-1.44	0.17

Supplemental Table 2 – Univariable Cox Proportional Hazards (CoxPH) regression of baseline immunity on late CMV Disease. Univariable Cox Proportional Hazards (CoxPH) regression of baseline (i.e., post-transplant day 100) immunity on late CMV Disease. “Concordance index” (i.e., C-index) analyses were used to optimize the predictive capability of each immune parameter on the development of endpoint adjudicated late CMV disease. Immune parameters were subsequently dichotomized according to the listed threshold cutoffs for CoxPH models.

CI= confidence interval, HR= hazard ratio.

[A]For neutralizing antibodies (nAb), a cutoff CMV nAb titer of 32 was selected based on previously published studies (see text for reference).

Immune Parameter	Threshold Quantile	Cutoff Value	Adjusted BH p-value
CD8 Polyfunctional IFN-γ T-cells	5%-60%	0 cells/uL	0.14
CD4 Polyfunctional IFN-γ T-cells	60%	0.06 cells/uL	0.10
CD8 Polyfunctionality Score	55%	0.04	0.27
CD4 Polyfunctionality Score	70%	0.08	0.14
CD8 Functionality Score	70%	0.18	0.14
CD4 Functionality Score	55%	0.08	0.20
$CD3^{neg}CD56^{bright}CD57^{neg}NKG2C^{pos}$ NK Cells	70%	0.85 cells/uL	0.14
$CD3^{neg}CD56^{dim}CD57^{neg}NKG2C^{pos}$ NK Cells	35%	0.54 cells/uL	0.05
$CD3^{neg}CD56^{dim}CD57^{pos}NKG2C^{pos}$ NK Cells	55%	0.32 cells/uL	0.05
Absolute Lymphocyte Count	75%	1270 cells/uL	0.14

Supplemental Table 3 – Benjamin Hochberg (BH) adjustment of multivariable Cox regression results.

	PC1	PC2	PC3	PC4	PC5	PC6	PC7	PC8	PC9	PC10	PC11
**Neutralizing Antibody Titer**	-0.1415	0.059418	0.742382	-0.55751	-0.21073	-0.21875	0.057905	-0.12258	-0.05315	0.02777	0.016373
**CD4 Functionality Score**	-0.42814	-0.09238	-0.21266	-0.13071	-0.05967	-0.05149	-0.13702	-0.33488	0.218446	-0.70498	0.253
**CD4 Polyfunctionality Score**	-0.39912	-0.15709	-0.29458	-0.19451	-0.02656	0.071764	-0.03849	-0.52859	0.075668	0.614487	-0.14611
**CD8 Functionality Score**	-0.42122	0.04533	0.106817	0.259615	-0.22125	-0.03757	-0.33878	0.198356	-0.14191	-0.13486	-0.70634
**CD8 Polyfunctionality Score**	-0.41837	0.050079	0.089989	0.287795	-0.22949	-0.03452	-0.27218	0.294026	-0.17189	0.278699	0.640253
**CD4 Polyfunctional T-cells**	-0.30144	-0.1781	-0.32627	-0.47947	0.294124	-0.21291	0.226362	0.545042	-0.24288	-0.00921	-0.043
**CD8 Polyfunctional T-cells**	-0.31465	-0.15229	0.157908	0.306218	-0.16529	0.274025	0.807345	0.005027	0.015802	-0.06293	-0.02547
**Absolute Lymphocyte Count**	-0.1901	-0.24912	0.395286	0.122211	0.768698	0.31478	-0.19424	-0.0444	0.016797	-0.00932	0.02365
**CD3^{neg}CD56^{bright}CD57^{neg}NKG2C^{pos} NK cells**	-0.05745	0.488918	-0.08742	-0.3325	-0.08234	0.76866	-0.06641	0.083048	-0.16272	-0.06197	0.014814
**CD3^{neg}CD56^{dim}CD57^{neg}NKG2C^{pos} NK cells**	-0.20729	0.540387	0.01975	0.029084	0.203227	-0.17134	0.099488	0.202434	0.723136	0.131859	-0.0439
**CD3^{neg}CD56^{dim}CD57^{pos}NKG2C^{pos} NK cells**	-0.11241	0.557373	-0.04109	0.184519	0.31129	-0.31944	0.180166	-0.34739	-0.53397	-0.05848	0.018363

Supplemental Table 4 – Loading values for individual immune parameters used for each principal component.

Marker	Fluorochrome	Vendor	Clone	Catalog Numbers
CD3	BUV395	BD Biosciences	UCHT1	563546
				624164
CD4	BUV496	BD Biosciences	SK3	564651
				624224
CD8	BUV805	BD Biosciences	SK1	564912
CD45RA	BUV737	BD Biosciences	HI100	564442
IFNγ	V450	BD Biosciences	B27	560371
NKG2C	Ax700	R & D Systems	134591	FAB138N-025
				FAB138N-100
PRF1	PE-Cy7	Biolegend	B-D48	353315
				353316
TNFA	FITC	eBioscience	MAb11	11-7349-82
IL2	PE	BD Biosciences	MQ1-17H12	559334
				624048
IL4	PerCP-Cy5.5	Biolegend	MP4-25D2	500821
				500822
CD154	APC	BD Biosciences	TRAP-1	560955
				555702
				624076
PD1	PE-Dazzle594	Biolegend	EH12.2H7	329939
				329940
CD14	BV605	Biolegend	M5E2	301833
				301834
live/dead	blue	Life Technologies		L23105
CD57	APC-Vio770	Miltenyi	REA769	130-111-966
				130-111-813
CCR7	BV785	Biolegend	G043H7	353229
				353230
CD56	BV650	BD Biosciences	NCAM16.2	564057

Supplemental Table 5 – Antibody types, sources, clone and catalog numbers used in this study.

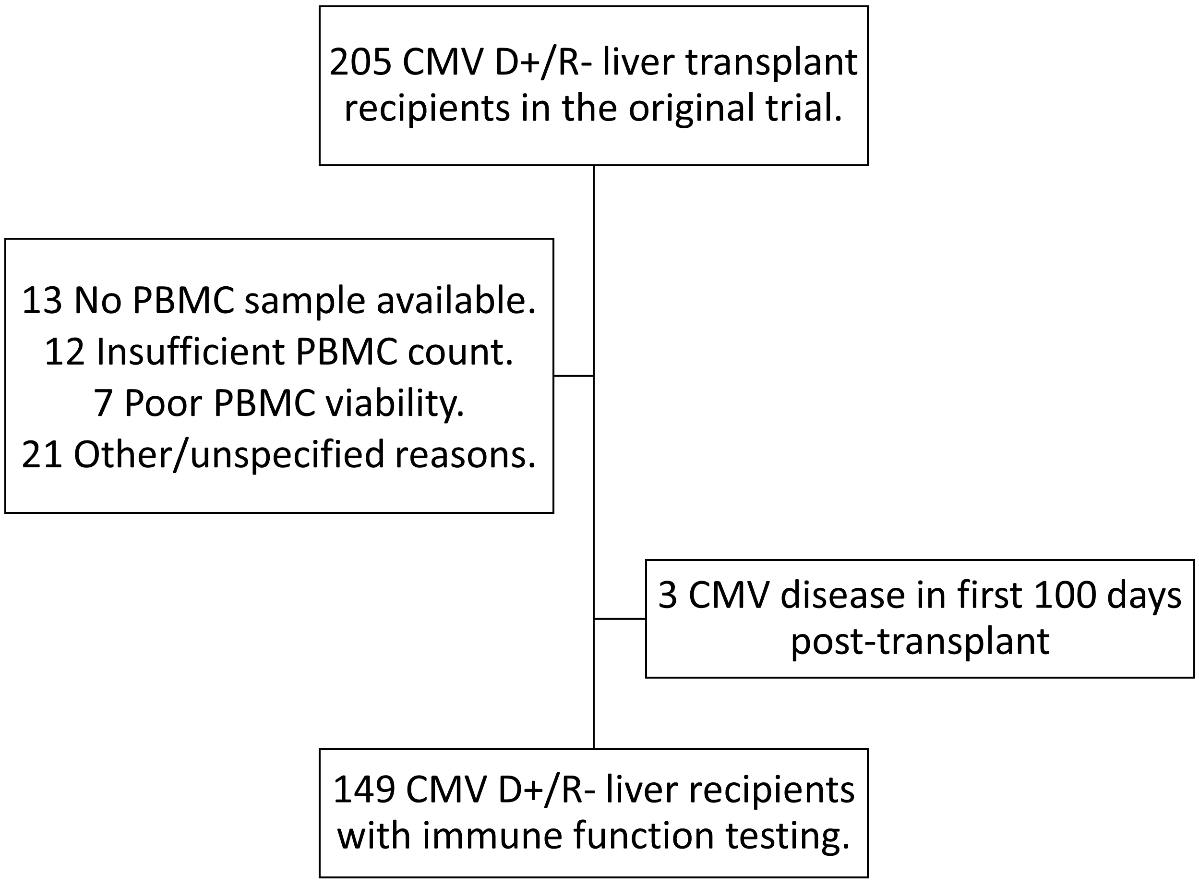

Supplemental Figure 1. – Patient sample and immune testing at 100 days post-transplant.

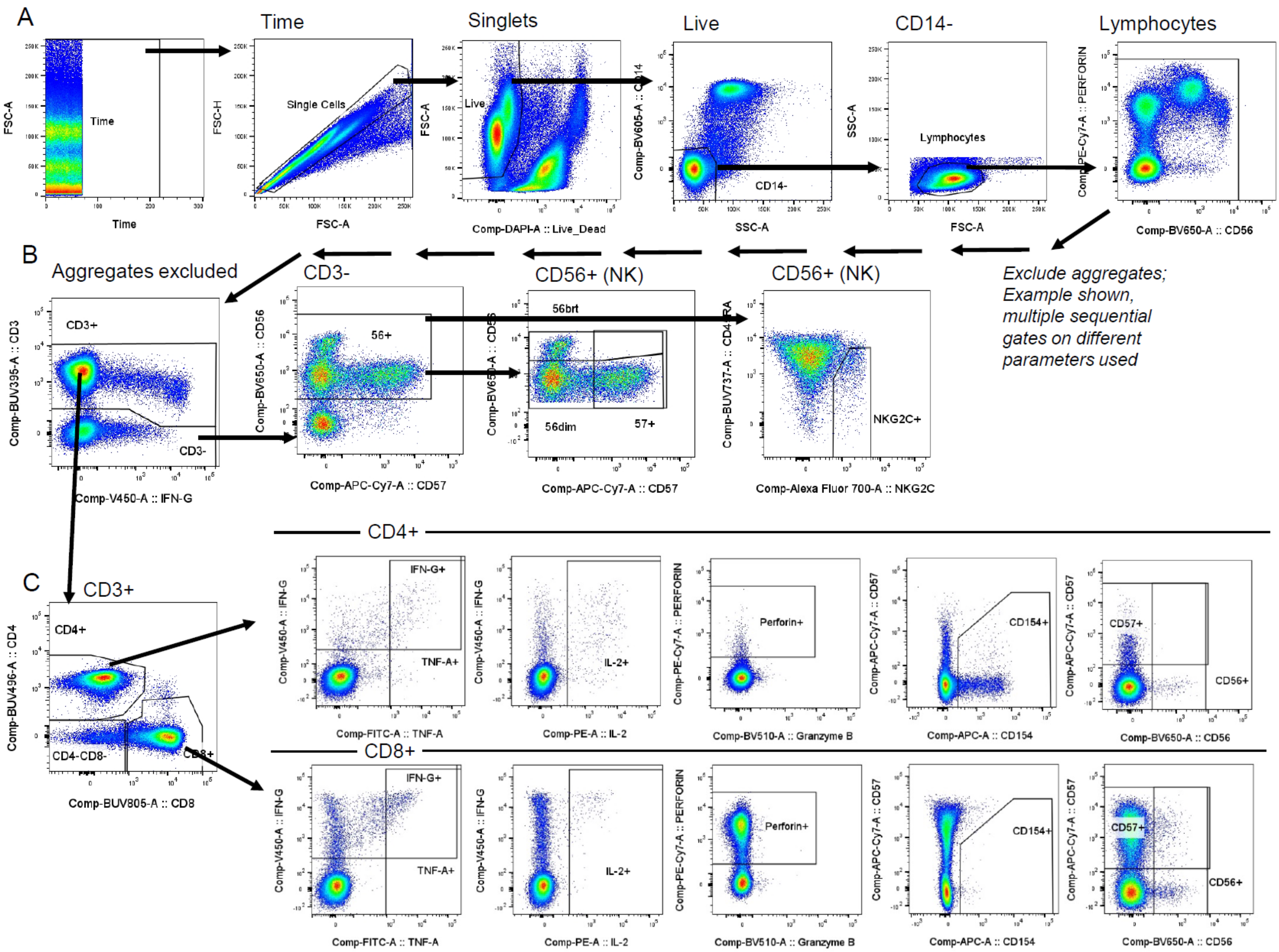

A
Time
Singlets
Live
CD14-
Lymphocytes
Single Cells
Lymphocytes
Exclude aggregates; Example shown, multiple sequential gates on different parameters used
B
Aggregates excluded
CD3-
CD56+ (NK)
CD56+ (NK)
CD3+
CD3-
56+
56brt
56dim
57+
NKG2C+
C
CD3+
CD4+
CD4-CD8-
CD8+
CD4+
IFN-G+
TNF-A+
IL-2+
Perforin+
CD154+
CD57+
CD56+
CD8+
FSC-A
Time
FSC-H
Comp-DAPI-A :: Live_Dead
SSC-A
Comp-BV650-A :: CD56
Comp-PE-Cy7-A :: PERFORIN
Comp-BUV395-A :: CD3
Comp-V450-A :: IFN-G
Comp-APC-Cy7-A :: CD57
Comp-Alexa Fluor 700-A :: NKG2C
Comp-BUV496-A :: CD4
Comp-BUV805-A :: CD8
Comp-FITC-A :: TNF-A
Comp-PE-A :: IL-2
Comp-BV510-A :: Granzyme B
Comp-APC-A :: CD154

Supplemental Figure 2. – Gating strategy. Gating strategy depicting a representative patient response to CMV pp65. **A.** First, a time gate was drawn to ensure stable collection of samples, followed by a singlet gate and dead cell exclusion gate. Cells in the "live" gate were restricted by CD14- gate to remove monocytes and then by size and granularity to identify lymphocytes, followed by sequential gates using different parameters to exclude aggregates, with one representative example shown. **B.** CD3- cells were then selected and subset by CD56+ to define NK cells, then either CD56^{bright} and ^{dim} and CD57, or NKG2C. **C.** CD3+ cells were selected (see first panel of **B**) and CD3+CD4 and CD3+CD8 T-cell subsets were gated for expression of functional markers and cytokines (IFNγ, TNFa, IL-2, perforin, and CD154). Note: granzyme B appears in the gating tree vs perforin but was ultimately not used in the panel. All markers were displayed and gated against the appropriate channel to allow optimum gating while avoiding potential spread from overlapping channels as identified by fluorescent minus one (FMO) controls, which may result in angled gates. Although not shown, functional gates were also placed in consideration of minimizing inclusion of background staining observed in DMSO controls, and were weighted towards specificity. Boolean combination gates were created as needed to interrogate subsets for both NK and T cells.

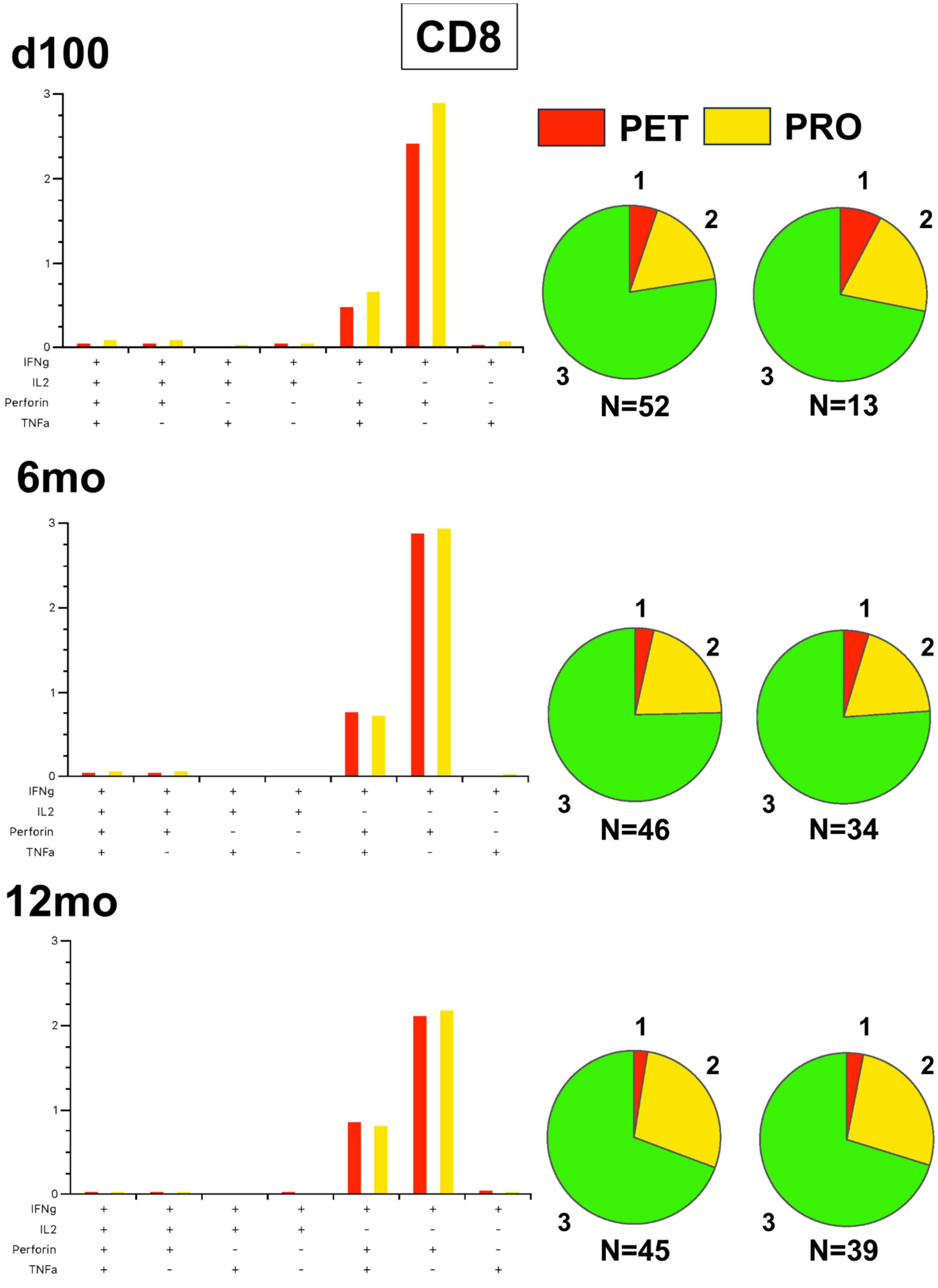
CD8
d100
6mo
12mo
PET
PRO
IFNg
IL2
Perforin
TNFa
1
2
3
N=52
N=13
N=46
N=34
N=45
N=39

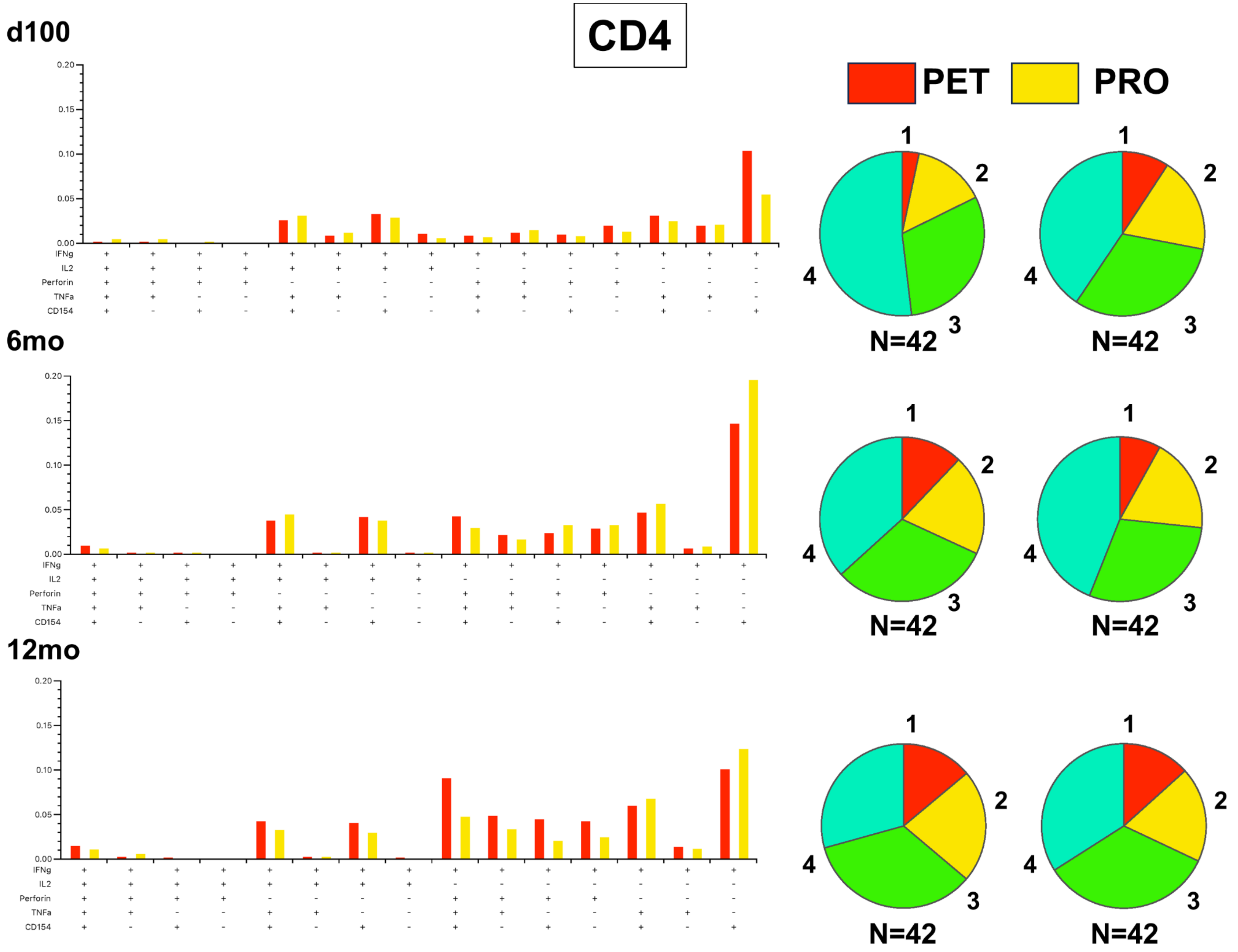

CD4
PET
PRO
d100
6mo
12mo
IFNg
IL2
Perforin
TNFa
CD154
N=42

Supplemental Figure 3. – Relative proportions of CMV-specific polyfunctional T-cells and combinations of functional markers based on the expression of IFNγ at 100 days, 6 months, and 12 months post-transplant. CMV-specific polyfunctional CD8 (Top) and CD4 (Bottom) T-cell subsets were categorized based on the number of functional markers expressed in addition to IFNγ. Specifically, the relative proportions of CD8 and CD4 T-cell immune responses expressing "IFNγ plus at least one additional measured functional marker" (i.e., TNFA, IL2, CD154, or PRF1) were measured in response to stimulation with CMV pp65 peptide library. CD154 and IL4 were removed from calculation of polyfunctional CD8 T-cell while IL4 responses were removed from calculation of polyfunctional CD4 T-cell responses given low expression in these cell compartments (see text for references). Immune responses were background subtracted using DMSO as negative control responses and positive responses were defined as T-cell frequencies greater than 0.05% above background and at least 3-fold greater than DMSO response in the same cell population. Only patients with positive polyfunctional responses are depicted in the figures above.

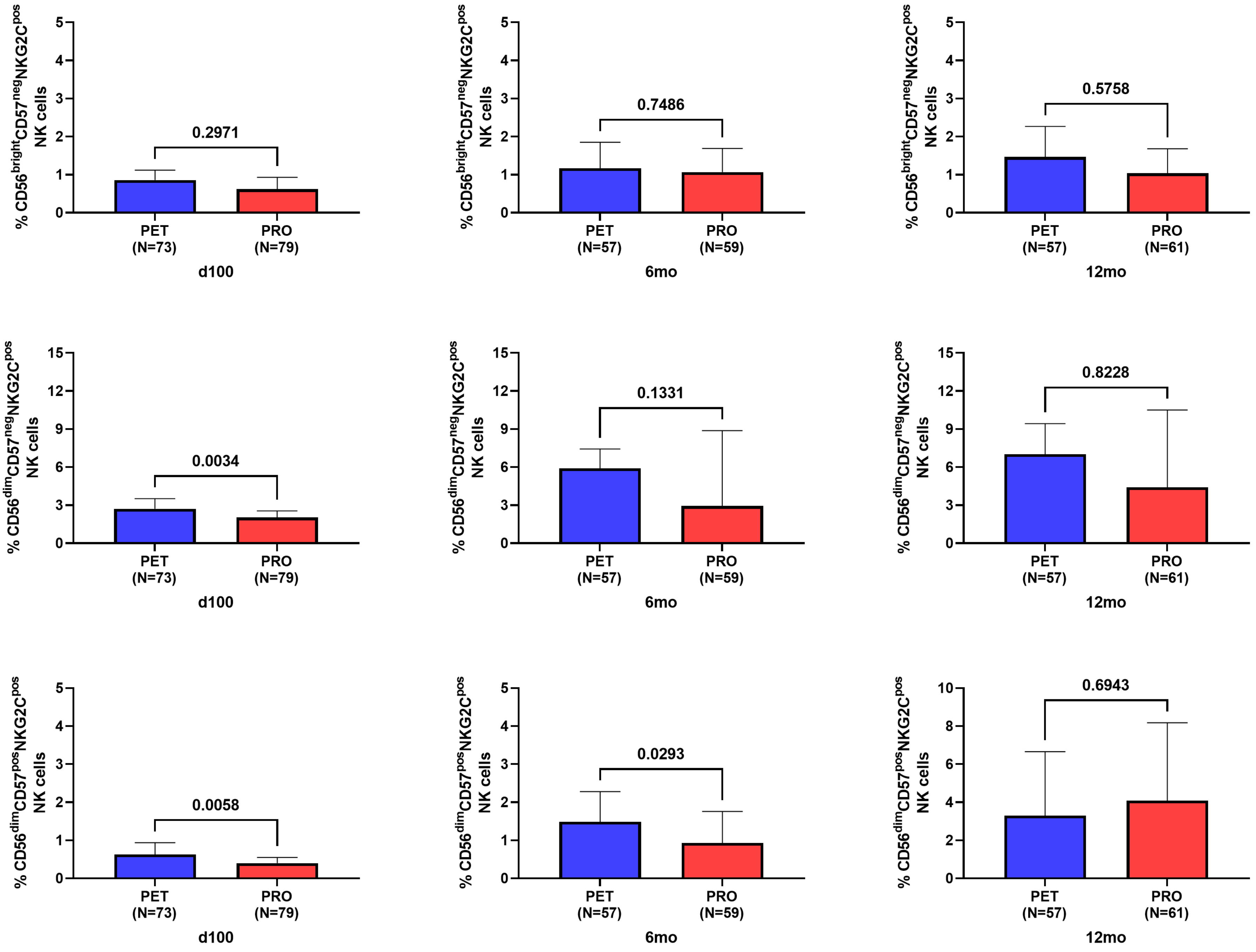
% CD56^{bright}CD57^{neg}NKG2C^{pos} NK cells
0.2971
PET (N=73)
PRO (N=79)
d100
0.7486
PET (N=57)
PRO (N=59)
6mo
0.5758
PET (N=57)
PRO (N=61)
12mo
% CD56^{dim}CD57^{neg}NKG2C^{pos} NK cells
0.0034
PET (N=73)
PRO (N=79)
d100
0.1331
PET (N=57)
PRO (N=59)
6mo
0.8228
PET (N=57)
PRO (N=61)
12mo
% CD56^{dim}CD57^{pos}NKG2C^{pos} NK cells
0.0058
PET (N=73)
PRO (N=79)
d100
0.0293
PET (N=57)
PRO (N=59)
6mo
0.6943
PET (N=57)
PRO (N=61)
12mo

Supplemental Figure 4. – Proportions of examined NKG2C-expressing NK Cell Subtypes at 100 days, 6 months, and 12 months post-transplant. NK cell subsets were categorized based on cell surface level expression of CD56 (i.e., bright vs dim) and CD57 (i.e., positive vs negative). Specifically, the proportions of $CD3^{neg}CD56^{bright}CD57^{neg}NKG2C^{pos}$, $CD3^{neg}CD56^{dim}CD57^{neg}NKG2C^{pos}$, and $CD3^{neg}CD56^{dim}CD57^{pos}NKG2C^{pos}$ NK cells are shown in PET vs PRO groups at all three time points. In boxplots, median cell proportions are shown with whiskers representing the 95% confidence interval. Comparisons were made using 2-sided Wilcoxon rank-sum testing at 95% confidence interval.

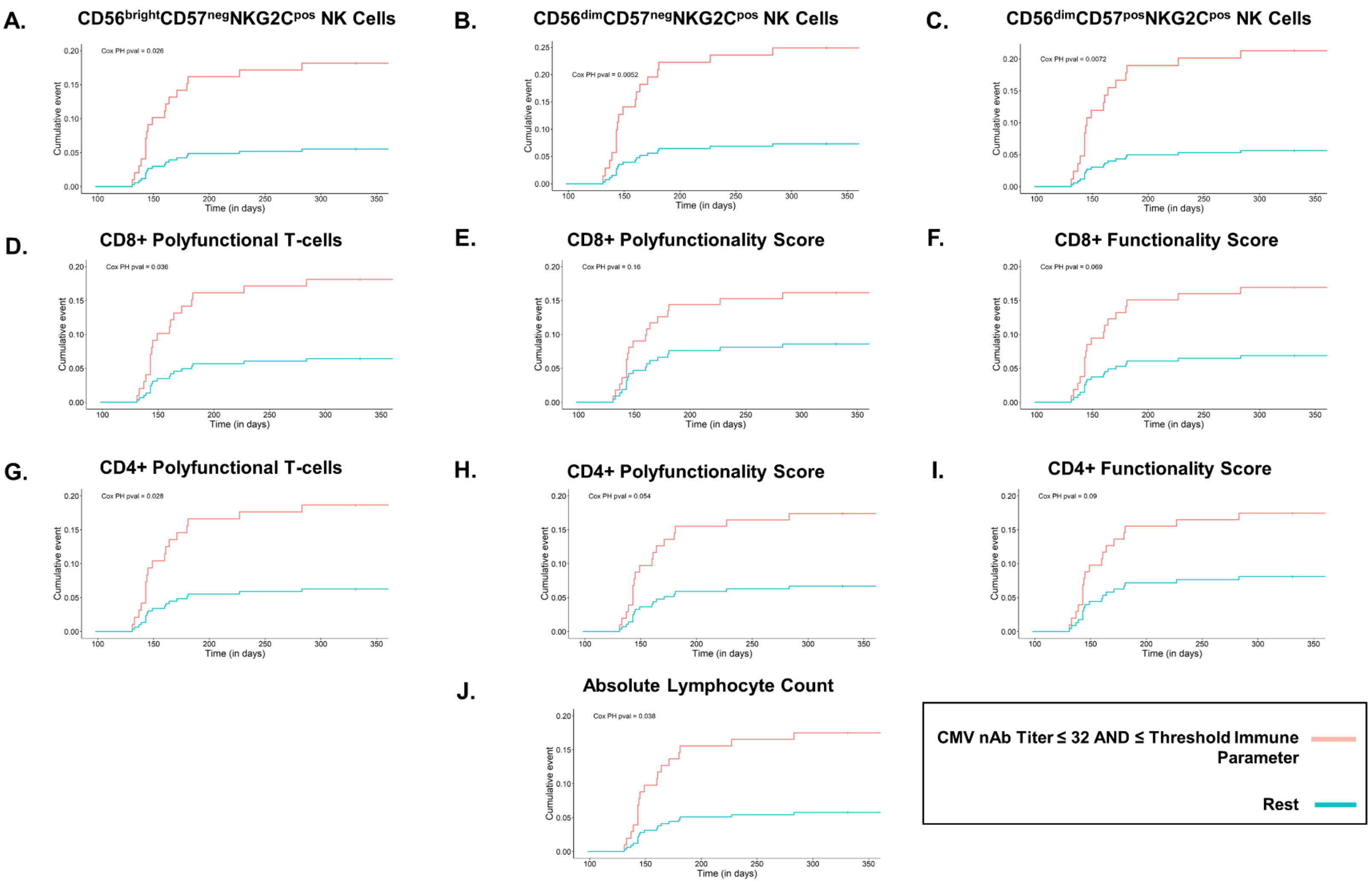

Supplemental Figure 5 – Cumulative incidence of late CMV disease following high-risk, CMV D+R- liver transplant in patients with baseline combined below threshold levels of CMV-specific T-cell and neutralizing antibody compared to all other patients. The cumulative incidence of endpoint adjudicated late CMV disease following high-risk, CMV D+R- liver transplant in patients with baseline neutralizing antibody (nAb) titers ≤ 32 combined with either low T-cell or NK cell immunity vs all other patients according to the dichotomous threshold cutoffs listed in

Table 2. Absolute counts of (**A**) $CD3^{neg}CD56^{bright}CD57^{neg}NKG2C^{pos}$, (**B**) $CD3^{neg}CD56^{dim}CD57^{neg}NKG2C^{pos}$, (**C**) $CD3^{neg}CD56^{dim}CD57^{pos}NKG2C^{pos}$ NK cells, (**D**) polyfunctional absolute CD8 T-cell counts, (**E**) CD8 polyfunctionality scores, (**F**) CD8 functionality scores, (**G**) polyfunctional absolute CD4 T-cell counts, (**H**) CD4 polyfunctionality scores, (**I**) CD4 functionality scores, and (**J**) absolute lymphocyte counts were stratified such that patients with baseline nAb titers ≤ 32 combined with either low T-cell or NK cell immunity were called as the reference group (red curves); whereas all other patients were placed into a separate comparator group (blue curves).

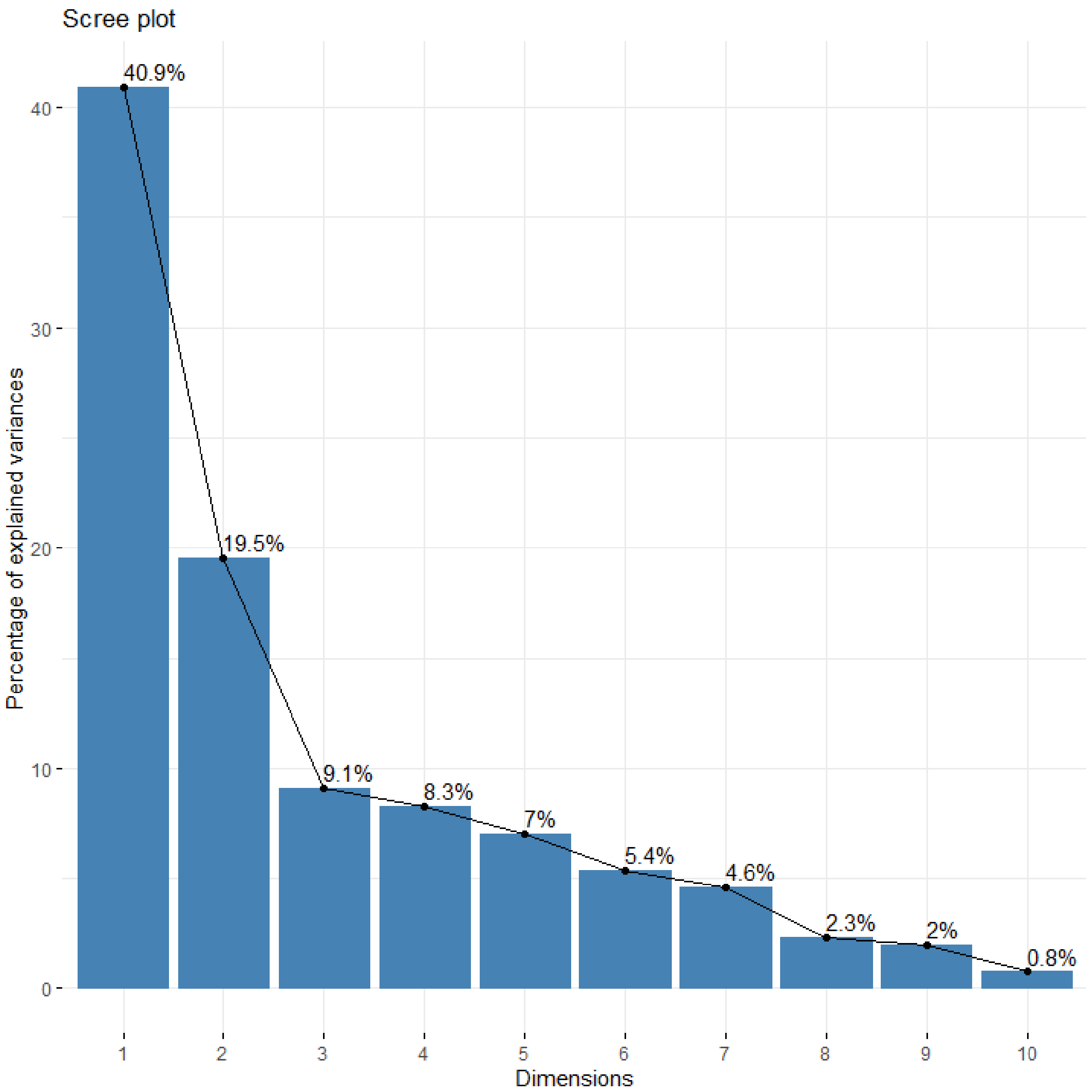

Supplemental Figure 6 – Scree plot of principal components. Scree plot of principal components (PCs) ordered from largest variance to smallest variance. Based on this plot, PC1 and PC2 account for 57.3% of the total variance in the data and were used to create correlation plots and in regression analyses were applicable.

1 Koelle DM, Corey L. Immunological herpes simplex virus antigens and methods for use thereof. US Patent 6,375,952. April 23, 2002.
2 Koelle DM, Chen H, Corey L, Hosken NA, McGowan P, Fling SP, Posavad CM. Immunologically significant herpes simplex virus antigens and methods for identifying and using same. US Patent 6,413,518. July 2, 2002.
3 Koelle DM, Hosken NA, Posavad CM, Chen H, McGowan P. Immunologically significant herpes simplex virus antigens and methods for using same. US Patent 6,814,969. November 9, 2004.
4 Koelle DM, Corey L Immunological herpes simplex virus antigens and methods for use thereof. US Patent 6,855,317. February 15, 2005.
5 Koelle DM, Chen H, Corey L Immunologically significant herpes simplex virus antigens and methods for identifying and using same. US Patent 6,962,709. November 8, 2005.
6 Koelle DM, Hosken NA, Posavad CM, Chen H, McGowan P. Immunologically significant herpes simplex virus antigens and methods for using same. US Patent 7,037,509. May 2, 2006.
7 Koelle DM, Liu Z, Corey L. Rapid, efficient purification of HSV-specific lymphocytes and HSV antigens identified via same. US Patent 7,078,041. July 18, 2006.
8 Koelle DM, Liu Z, Corey L. Rapid, efficient purification of HSV-specific lymphocytes and HSV antigens identified via same US Patent 7,431,934. October 7, 2008.
9 Vilalta A, Margalith M, Dong L, Koelle DM. Compositions and methods for vaccinating against HSV-2. US Patent 7,628,993. December 8, 2009.
10 Koelle DM, Liu Z, Corey L. Rapid, efficient purification of HSV-specific T-lymphocytes and HSV antigens identified via same US patent 7,666,434. Feb. 23, 2010.
11 Koelle DM, Liu Z, Corey L. Rapid, efficient purification of HSV-specific T-lymphocytes and HSV antigens identified via same US patent 7,744,903. June 29, 2010.
12 Vilalta A, Margalith M, Dong L, Koelle DM. Compositions and methods for vaccinating against HSV-2. US Patent 7,897,339. Feb. 1, 2011.
13 Vilalta A, Margalith M, Dong L, Koelle DM. Compositions and methods for vaccinating against HSV-2. US Patent 7,935,352. May 3, 2011.
14 Koelle DM, Corey L. Immunological herpes simplex virus antigens and methods for use thereof. US Patent 8,067,010. Nov. 29, 2011.
15 Koelle DM, Liu Z, Corey L. Rapid, efficient purification of HSV-specific T-lymphocytes and HSV antigens identified via same US patent 8,197,824. June 12, 2012.
16 Vilalta A, Margalith M, Dong L, Koelle DM. Compositions and methods for vaccinating against HSV-2. US Patent 8,263,087. Sept. 11, 2012.
17 Vilalta A, Margalith M, Dong L, Koelle DM. Compositions and methods for vaccinating against HSV-2. US Patent 8,293,248. Oct. 23, 2012.
18 Koelle DM, Jing L. HSV-1 epitopes and methods for using same. US Patent 8,460,674. July 11, 2013.
19 Vilalta A, Margalith M, Dong L, Koelle DM. Compositions and methods for vaccinating against HSV-2. US Patent 8,828,408. Sept. 9, 2014.
20 Vilalta A, Margalith M, Dong L, Koelle DM. Compositions and methods for vaccinating against HSV-2. US Patent 8,834,894. Sept. 16, 2014.
21 Vilalta A, Margalith M, Dong L, Koelle DM. Compositions and methods for vaccinating against HSV-2. US Patent 8,840,903. Sept. 23, 2014.
22 Vilalta A, Margalith M, Dong L, Koelle DM. Compositions and methods for vaccinating against HSV-2. US Patent 8,840,904. Sept. 24, 2014.
23 Koelle DM, Corey L. Immunological herpes simplex virus antigens and methods for use thereof. US Patent 8,852,602. Oct.7, 2014.
24 Vilalta A, Margalith M, Dong L, Koelle DM. Compositions and methods for vaccinating against HSV-2. US Patent 8,852,610. Oct. 7, 2014.

25 Vilalta A, Margalith M, Dong L, Koelle DM. Compositions and methods for vaccinating against HSV-2. US Patent 8,852,611. Oct. 7, 2014.
26 Corey L, Laing K, Wald A, Koelle DM. Antigenic peptide of HSV-2 and methods for using same. US Patent 9,044,447. June 2, 2015.
27 Koelle DM, Liu Z, Corey L. Rapid, efficient purification of HSV-specific lymphocytes and HSV antigens identified using same. US Patent 9,138,473. Sep. 22, 2015.
28 Vilalta A, Margalith M, Dong L, Koelle DM. Compositions and methods for vaccinating against HSV-2. US Patent 9,161,973. Oct 20, 2015.
29 Vilalta A, Margalith M, Dong L, Koelle DM. Compositions and methods for vaccinating against HSV-2. US Patent 9,205,146. Dec. 8, 2015.
30 Koelle DM, Verjans GMGM. HSV-1 epitopes and methods for using same. US Patent 9,328,144. May 3, 2016.
31 Corey L, Laing KJ, Wald A, Koelle DM. Antigenic peptide of HSV-2 and methods for using same. US patent 9,579,376. February 28, 2017.
32 Koelle DM, Liu Z, Corey L. Rapid, efficient purification of HSV-specific T-lymphocytes and HSV antigens identified via same. US Patent 9,675,688. June 13, 2017.
33 Chapuis AG, Nghiem PT, McAfee MS, Miller NJ, Paulson KG, Koelle DM, Schmitt TM, Church C. High affinity Merkel cell polyomavirus T antigen-specific TCRs and uses thereof. US Patent 11,534,461 Dec 27, 2022.
